# Framework Mutations of the 10-1074 bnAb Increase Conformational Stability, Manufacturability, and Stability While Preserving Full Neutralization Activity

**DOI:** 10.1016/j.xphs.2019.07.009

**Published:** 2020-01

**Authors:** Bruce A. Kerwin, Chelsey Bennett, Yan Brodsky, Rutilio Clark, J. Alaina Floyd, Alison Gillespie, Bryan T. Mayer, Megan McClure, Christine Siska, Michael S. Seaman, Kelly E. Seaton, Jeremy Shaver, Georgia D. Tomaras, Nicole L. Yates, Randal R. Ketchem

**Affiliations:** 1 Just Biotherapeutics, Inc., Department of Design, 401 Terry Avenue N., Seattle, Washington 98109; 2 Fred Hutchinson Cancer Research Center, Division of Vaccine and Infectious Disease, 1100 Fairview Avenue N., Seattle, Washington 98109; 3 Center for Virology and Vaccine Research, Beth Israel Deaconess Medical Center, 3 Blackfan Circle, E/CLS-1001, Boston, Massachusetts 02115; 4 Departments of Surgery, Immunology, Molecular Genetics and Microbiology, Duke Human Vaccine Institute, Duke University, Durham, North Carolina 27710

**Keywords:** analytical biochemistry, antibody(s), biopharmaceutical characterization, developability, fluorescence spectroscopy, high throughput technology(s), HIV/AIDS, pharmacokinetics, physical stability, protein aggregation, protein folding, protein structure, stability, stabilization, bnAbs, broadly neutralizing antibodies, CDR, Complementary determining region, COGs, Cost of goods, DSF, Differential scanning fluorimetry, Gdn-HCl, Guanidine hydrochloride, HC, Heavy chain, HMW, High molecular weight, HP-SEC, High performance size exclusion chromatography, LC, Light chain, mAb, monoclonal antibody, PK, Pharmacokinetic, PBS, Phosphate buffered saline, PEG, Polyethylene glycol, CDCA, Stability design by covariance analysis

## Abstract

The broadly neutralizing anti-HIV antibody, 10-1074, is a highly somatically hypermutated IgG1 being developed for prophylaxis in sub-Saharan Africa. A series of algorithms were applied to identify potentially destabilizing residues in the framework of the Fv region. Of 17 residues defined, a variant was identified encompassing 1 light and 3 heavy chain residues, with significantly increased conformational stability while maintaining full neutralization activity. Central to the stabilization was the replacement of the heavy chain residue T108 with R108 at the base of the CDR3 loop which allowed for the formation of a nascent salt bridge with heavy chain residue D137. Three additional mutations were necessary to confer increased conformational stability as evidenced by differential scanning fluorimetry and isothermal chemical unfolding. In addition, we observed increased stability during low pH incubation in which 40% of the parental monomer aggregated while the combinatorial variant showed no increase in aggregation. Incubation of the variant at 100 mg/mL for 6 weeks at 40°C showed a 9-fold decrease in subvisible particles ≥2 μm relative to the parental molecule. Stability-based designs have also translated to improved pharmacokinetics. Together, these data show that increasing conformational stability of the Fab can have profound effects on the manufacturability and long-term stability of a monoclonal antibody.

## Introduction

With more than 30 years of the HIV epidemic, there is still no cure or an effective vaccine for HIV. In 2017, there were an estimated 36.9 million people living with HIV worldwide, with almost 2 million new infections each year.^1^ Sub-Saharan Africa is home to only 16.6% of the global population,^2^ yet accounts for 70% of the global burden of HIV infection.^1^ Multiple strategies have been adopted to help reduce the rate of transmission including educational campaigns promoting safer sexual practices, expanded HIV testing, male circumcision, and prescribing antiretroviral drugs for preexposure prophylaxis.^3^, ^4^, ^5^ Of these strategies, preexposure prophylaxis using the combination drug, tenofovir disoproxil fumarate (TDF)/emtricitabine, is the most effective with a prevention rate exceeding 90% when taken as prescribed.^3^ For the drug, Truvada®, the prescribing information indicates dosage of a single pill taken every day. Lower rates of HIV prevention have been observed in a number of studies and linked to the degree of adherence. Other strategies are clearly necessary for effective control and reduction of the epidemic.

Since 2010 with the advent of single-cell antibody cloning techniques, individuals have been identified who produce antibodies capable of binding the gp120 spike protein trimer^6^, ^7^ and neutralizing a broad array of HIV variants. These broadly neutralizing antibodies (bnAbs), or bnAbs, are highly somatically mutated, evolving in a small number of individuals several years after the initial infection.^8^, ^9^, ^10^ Four primary regions of the gp120 spike protein on the surface of the HIV are targeted by the bnAbs^11^, ^12^, ^13^ and include the membrane proximal region, the CD4 binding site, V1/V2 glycan region, and V3 glycan. Unlike most antibodies that primarily demonstrate somatic mutation in the complementarity-determining region (CDR), bnAbs also have multiple mutations in the framework region allowing for their increased breadth and potency.^8^ In addition, many bnAbs have extended CDR3 loops, allowing for the V1/V2 region-binding bnAbs and the V3 region bnAbs to bind both the glycan shield of the gp120 and the underlying amino acid structure.

Since their discovery, a handful of bnAbs have been used in human clinical trials for reducing viremia in infected individuals. The bnAbs VRC01,^14^ 3BNC117,^15^ and 10-1074^16^ have all demonstrated a reduction of viremia of up to 2.5 logs after a single dose of up to 30 mg/kg. In addition, studies with nonhuman primates have demonstrated that a single 20-mg/kg dose of either VRC01, 3BNC117, or 10-1074 protected the animals against viral infection during weekly low-dose simian/human immunodeficiency virus challenges for up to 23 weeks.^17^ From these studies and many others, the HIV community has evolved the idea of using bnAbs as a prophylactic tool against the spread of HIV. To commercialize the bnAbs, they must be produced in large quantities at relatively low cost of goods (COGs). While COGs can be severely reduced using small-scale and high-capacity flexible facilities,^18^ the antibodies must also be sequence optimized for manufacturability because even minor changes can affect productivity and ease of purification.^19^, ^20^

Multiple publications have demonstrated that the amino acid sequence of the constant and variable regions of antibodies can affect molecular stability, expression levels, downstream processing conditions, and formulation conditions required for stable long-term storage.^19^, ^20^, ^21^, ^22^, ^23^, ^24^, ^25^, ^26^, ^27^ Clark et al.^20^ demonstrated that introduction of multiple amino acid substitutions into the Fv region of an IgG2 to alter the amount of exposed hydrophobic and hydrophilic surface areas resulted in an increased titer that correlated with an increase in the thermal stability of the variants. Pettit et al.^22^ inserted a missing Lys residue at position 148 of the light chain (LC) for an anti-ebola antibody and demonstrated a significant increase in titer, reduction in high-molecular-weight (HMW) species, and increase in thermal stability by differential scanning fluorimetry (DSF) from material produced by stable CHO cell lines as compared to the parental material demonstrating the importance of primary sequence in expressions and biophysical properties of an antibody.

In the work presented here, we were interested in sequence optimizing the 10-1074 bnAb for increased conformational stability, hypothesizing that this would lead to better manufacturability and storage parameters. This bnAb is of particular interest because it exhibits unusual potent neutralization, including broad reactivity against newly transmitted viruses,^28^ and thus could potentially be used in combination with other bnAbs for prophylaxis against HIV transmission^29^, ^30^ in Sub-Saharan Africa, necessitating low COGs and increased long-term stability outside the cold chain for storage and distribution. Because of the large number of potentially destabilizing sites we identified in the Fv region of the antibody, we developed a dual-stage approach for the molecular optimization procedure. Focusing on conformational stability by DSF using an information-rich metric, the weighted shoulder score (WSS), enabled the identification of 5 sites that retained activity and showed improvement as single mutations. Combination of these sites allowed for identification of multiple variants with significant increases in conformational stability as measured by both thermal and chemical methods. In addition, molecules with both increased thermal and chemical stability demonstrated improvements during low-pH treatment and in long-term stability at 40°C, both factors which are important for improved manufacturability and storage time outside of the cold chain, an important factor for supplying these drugs to the Sub-Saharan Africa. In addition, production of stable CHO cell lines with one of the variants has consistently resulted in productivity levels of >2 g/L/d, allowing for production in flexible manufacturing facilities at low cost.

## Materials and Methods

### Calculation of Covariance Violations

Mutational impacts on stability were calculated using Stability Design by Covariance Analysis which uses a method known as covariance analysis^31^ to evaluate all possible residue pairs in an underlying antibody sequence database to search for specific residue positions that covary, meaning their residue properties for those sites tend to be correlated. This correlation was based not on specific residue types but on residue groupings of acidic (DE), basic (KRH), hydrophobic (ALIMCVP), aromatic (FWY), neutral (NQSTBZ), and deletion (G*X). Positions for the 10-1074 were assigned based on the AHo^32^ numbering system. Positions that break the covariance with multiple covarying states within the underlying reference set are considered to be covariance violators and potential stability-degrading sites. 10-1074 contains 17 strong covariance violations (Table 1). There were also one potential isomerization site and one potential deamidation site which could impact the neutralization function. In addition, 4 missing residues that normally present in the N-terminal peptide were identified and included in a subset of the variant design. Structure considerations, structurally aligned^32^ positional frequency analysis, and germline background were used to determine modifications that would repair these violations. Building all possible combinatorial variants with a single remediation at each site would have resulted in 524,287 variants of the parental antibody. To reduce the variant production set, the variant space was explored in 2 rounds of engineering. The initial round included all the sites as individual site variants, one variant with all the sites deemed potentially structurally safe for maintained activity, one variant grouping the sites structurally near the LC N-terminal region, and one variant grouping the sites sequentially near the LC N-terminal region, resulting in 22 variants. Activity and biophysical characterization data from the initial round were then used to downselect the positions to be combined in a second engineering round. This resulted in 5 positions combined in all combinations not explored in the initial round, resulting in 26 variants.

**Table 1:** 10-1074 Residues Identified as Potential Destabilizing and Variant of Each Site for Production

Molecule Set^a^	Light Chain	Heavy Chain
MS-194^b^		
MS-203	LmdV:Y2P	
MS-204	LmdV:R7P	
MS-205	LmdV:P9S	
MS-206	LmdV:E17Q	
MS-207	LmdV:H46Q	
MS-208	LmdV:P81.1N	
MS-209	LmdV:I81.3S	
MS-210	LmdV:N82G	
MS-211	LmdV:R88T	
MS-212	LmdV:D110E	
MS-213	LmdV:A142G	
MS-214		HV:D29G
MS-215		HV:S47P
MS-216		HV:V79T
MS-217		HV:R82V
MS-218		HV:L89F
MS-219		HV:T108R
MS-220		HV:K141Q
MS-224		HV:*N*75Q
MS-221	LmdV:Y2P LmdV:R7P LmdV:P9S	
MS-222	LmdV:Y2P LmdV:R7P LmdV:P9S LmdV:A142G	
MS-223	LmdV:Y2P LmdV:R7P LmdV:P9S LmdV:E17Q LmdV:H46Q LmdV:A142G	HV:S47P HV:K141Q
MS-257	LmdV:Gap5L LmdV:Gap6T LmdV:R7Q LmdV:Gap8P LmdV:Gap10S	
MS-258	LmdV:Gap5L LmdV:Gap6T LmdV:Gap8P LmdV:Gap10S	
MS-259	LmdV:Gap5L LmdV:Gap6T LmdV:R7Q LmdV:Gap8P LmdV:P9L LmdV:Gap10S	
MS-260	LmdV:Gap5L LmdV:Gap6T LmdV:Gap8P LmdV:P9L LmdV:Gap10S	

a All variants including the MS-194 contain the Xtend mutation (LS) in the Fc of the heavy chain.

b MS-194 is also referred to as parental (+LS) in the manuscript.

In addition to the mutations to increase conformational and colloidal stability, it was desirable to increase half-life, and therefore, the Xencor Xtend mutation set consisting of Fc-C:M104L and Fc-C:N112S was introduced to all variants in both the first and second rounds. As controls, the parental (+LS) molecule also contains the Xtend mutations, while the parental (-LS) does not.

### Production of Antibodies

Antibody materials were cloned and produced as previously described.^33^, ^34^ BnAb antibody materials were generated from transient expression of 2 suspension cell lines, human embryonic kidney 293 (HEK293; Syngene International, Bangalore, India) and Chinese hamster ovary (CHO-S; ExpiCHO from Thermo Fisher Scientific, Waltham, MA). The pTT5 mammalian expression vectors containing either a LC or heavy chain (HC) coding region were cotransfected into HEK293 cells at a viable cell density of 1*10^∧^6 cells/mL using polyethyleneimine and then 2-fold diluted with prewarmed medium to one-fifth of shake flask volume. Expression duration was 5-7 days at 37°C, 5% CO_2_, and 85% humidity at a shaking speed of 130 RPM with an orbit of 19 mm. The ExpiCHO-S^TM^ “max titer” method was followed essentially as described by Thermo Fisher Scientific (catalog number A29133, document part number A29518). The pcDNA3.4 expression vectors containing either LC or HC coding regions were cotransfected into CHO-S cells at a viable cell density of 6*10ˆ6 using expifectamine. The expression duration was 12 days at 32°C, 5% CO_2_, and 85% humidity at a shaking speed of 130 RPM with an orbit of 19 mm. All clarified supernatants were produced by pelleting the cells at 3000 g for 20 min followed by 0.22-μm filtration. Antibodies were purified from the clarified supernatants using MabSelect SuRe protein A resin. A sodium phosphate, sodium chloride buffer system with an arginine wash and an acetate pH 3.5 elution was used. Protein A elutions were neutralized with tris buffer exchanged into 20-mM sodium phosphate, 150 mM NaCl, pH 7.4.

### Neutralization Assays

Virus neutralization was evaluated using a luciferase-based assay in TZM.bl cells as previously described.^35^, ^36^ MAb samples were tested using a primary concentration of 25 μg/mL with 5-fold dilution series against a selected panel of Tier 2 HIV-1 Env pseudoviruses with a known sensitivity to 10-10,74.^7^ The IC_50_ and IC_80_ titers were calculated as the mAb concentration that yielded a 50% or 80% reduction in relative luminescence units, respectively, compared with the virus control wells after subtraction of cell control relative luminescence units. All assays were performed in a laboratory compliant with Good Clinical Laboratory Practice procedures.

### High-performance Size Exclusion Chromatography

High-performance size exclusion chromatography (HP-SEC) separates proteins based on differences in their hydrodynamic volumes. Molecules with larger hydrodynamic protein volumes elute earlier than molecules with smaller volumes. Undiluted samples were loaded onto a Waters XBridge Protein BEH SEC 200A column (3.5 μm, 7.8 × 300 mm) separated isocratically with a 100-mM sodium phosphate, 250-mM sodium chloride, pH 6.8 running buffer, and the eluent was monitored by ultraviolet absorbance at 220 nm. Purity was determined by calculating the percentage of each separated component as compared with the total integrated area.

### Differential Scanning Fluorimetry

Thermal transition temperatures by DSF were measured according to the method previously described.^37^ The analysis was carried out in phosphate buffered saline (PBS) buffer (20-mM sodium phosphate and 150-mM sodium chloride pH 7.1) at a final protein concentration of 0.15 mg/mL and a final Sypro Orange concentration of 3×. Protein and Sypro Orange were mixed at a 1:1 volumetric ratio in a 96-well PCR plate and analyzed using a Roche LightCycler 480 instrument equipped with a Thermal Shift Analysis Software program. Thermal curves were generated by heating the samples from 20°C to 95°C at a ramp rate of 4.4°C/s and 10 acquisitions per °C, at Ex = 465 nm and Em = 580 nm. Transition temperatures and shoulder scores were determined using the software supplied with the instrument based on the first derivative of the melting curve.

The WSS is calculated by first identifying the lowest temperature transition peak (T1) from the first derivative of the melting curve and normalizing the entire derivatized thermogram to the peak intensity of that T1 transition. A weighted sum of all nonnegative values above this temperature is calculated, where each normalized intensity value is multiplied by the squared difference in temperature between T1 and the given point. That is, the normalized intensity value for point *i* in the thermogram, I_i,norm_, is multiplied by a weight, W_i_, determined from:

$$W_{i}=\{\begin{array}{cc} {\left(T_{i}-T_{T1}\right)}^{2} & \mathrm{if}\left(T_{i}>T_{T1}\;\mathrm{and}\;I_{i,\mathrm{norm}}>0\right)\\ 0 & \mathrm{otherwise} \end{array}$$

where T_i_ is the temperature of the given intensity point and T_T1_ is the temperature of the T1 transition (inflection point in the original curve, peak of the derivative curve). WSS is then calculated summing over all points in the thermogram and taking the square root:

$$\mathrm{WSS}=\sqrt{\sum I_{i,\mathrm{norm}}W_{i}}$$

### Low pH Stability

The pH of protein samples at 1 mg/mL in PBS was lowered to approximately 3.3 using 2 M acetic acid. After a 30-min incubation, samples were neutralized to approximately pH 5 using 2 M tris base. Samples were measured for HMW species using the HP-SEC method and measured in duplicate. As a control, protein samples had PBS added that was the same volume of the 2 M acetic acid and 2 M Tris base and measured for HMW species.

### Relative Solubility

Solubility was assessed according to the method previously described.^38^ Analysis was performed in PBS and a final polyethylene glycol (PEG) 10,000 concentration of 7.9%. Protein at 1 mg/mL was diluted into the PEG solution at a 1:4 ratio and incubated at room temperature overnight in a 96-well 0.22-μm filter plate. After PEG incubation, samples are passed through the filter by centrifugation, and the remaining soluble protein is measured by a protein A titer assay.

### Chemical Unfolding

The chemical unfolding assay was completed as described by Floyd et al.^39^ Briefly, 32 guanidine hydrochloride (Gnd-HCl) concentrations in PBS ranging from 0 to 6 M Gnd were prepared using a Tecan Freedom EVO liquid-handling robot (Tecan Group Ltd.). Then, the protein samples at 1 mg/mL PBS were transferred to each Gnd-HCl concentration to achieve a final protein concentration of 0.05 mg/mL. After a 24-h incubation, the samples were measured on a SpectraMax M5 plate reader (Molecular Devices, LLC) (excitation: 280 nm, emission: 300-450 nm). The measured fluorescence intensity at 373 nm was corrected for scattering and stray light by subtraction of a small amount of the summed intensity measured between 300 and 320 nm (used as a surrogate for signal due to scattering) and then normalized to the total intensity measured between 320 and 440 nm to correct for total intensity fluctuations. Then, the chemical unfolding curve was generated by graphing each corrected intensity against the Gnd-HCl concentration. The inflection point of the curve was calculated and reported for each protein sample from this curve. Samples were completed in triplicate.

### Purification and Viral Inactivation

The performance of the combinatorial variants was investigated in a standardized purification model using material produced by transient expression in a CHO-S system. After a 14-day bioreactor production, the culture was harvested by centrifugation and depth filtration to remove cells and cellular debris. The cell culture supernatants were then applied to a MabSelect SuRe™ protein A column (GE Lifesciences), and the products eluted with a low-pH buffer. A small volume (2 mL) of each protein A eluate was titrated with acid to pH 3.5 and held for 60 min to evaluate variant stability in the low-pH viral inactivation unit operation. The remaining protein A elution pools were titrated to pH 5.0 and processed by cation exchange chromatography using a Fractogel® EMD SO_3_⁻ column. Bound product was eluted using a linear gradient of sodium chloride. The resulting CEX product pools were formulated into an acetate buffer at pH 5.2 with 9% sucrose.

### Accelerated Stability

Stability during storage of the molecules was assessed in an acetate buffer at pH 5.2 with 9% sucrose. Molecules were buffer exchanged and concentrated to 100 mg/mL using an Amicon centrifugal filtration unit with a 30-kD molecular weight cutoff. Polysorbate 80 was spiked into the solution to a final concentration of 0.01% (w/v). Samples were filtered using a 0.22-μm Pall Acrodisc into a sterile vial. Vials were capped and crimped and stored at 40°C for the duration of the study. At each time point, the vial was uncapped in a sterile hood and 10 μL of sample was removed for HP-SEC analysis as described previously. Vials were capped and crimped again and returned to 40°C storage. At select time points, 150 μL was removed for FlowCam analysis.

### Subvisible Particle Analysis

Subvisible particles were measured using a FlowCam 8100 benchtop microflow imaging system equipped with an 80-μm flow cell and a 10× magnification lens and controlled by the Visual Spreadsheet software (FluidImaging Technologies). Samples were equilibrated to room temperature and gently swirled to mix thoroughly. Single readings of 100 μL per sample were collected, and total particle concentration above 2 μm was recorded.

### Polyreactivity

Polyreactivity was carried out as previously described by Wardemann et al.^40^

### PK Analysis

#### Mouse Studies

Thirty-six 6- to 8-week-old B6.Cg-F*cgrt*^tm1Dcr^ Tg(CAG/FCGRT)276Dcr/DcrJ (Tg276, JAX stock#004919) male mice were transferred to the JAX animal facility. The Tg276 mice were homozygous for the mouse FcRn knockout and hemizygous for the human FcRn transgene. The mice were ear notched for identification and housed in individually and positively ventilated polysulfone cages with high-efficiency particulate air filtered air at a density of 4 mice per cage. The animal room was lighted entirely with fluorescent lighting, with a controlled 12-h light and dark cycle (6 AM to 6 PM light). The normal temperature and relative humidity ranges in the animal rooms were 22 ± 4°C and 50 ± 15%, respectively. The animal rooms were set to have 15 air exchanges per hour. Filtered tap water, acidified to a pH of 2.5 to 3, and standard laboratory chow was provided ad libitum.

The mice were distributed into groups of 4 per antibody molecule. Body weights were measured within 1 day of antibody administration. At 0 h, test antibodies were administered as intravenous injections in the tail vein at 10 mg/kg. Blood samples were collected from each mouse at 1 h, 8 h, 1 day, 2 days, 5 days, 7 days, 10 days, 14 days, 21 days, and 28 days. Aliquots (25 μL) were collected with K_3_EDTA as an anticoagulant. The blood samples were processed to plasma. Aliquots (10 μL) were diluted 1:10 with 50% glycerol in PBS, flash frozen in specialized 96-well storage plates and stored at −20°C. The plasma samples arrayed in the 96-well plates were shipped to the Duke University for enzyme-linked immunosorbent assay analysis.

Serum 10-1074 IgG levels were measured by anti-idiotype (ID) enzyme-linked immunosorbent assay^29^, ^41^ which has been validated for use in human samples and further developed for accurate and sensitive detection of infused 10-1074 mAb variants in mice. Briefly, high-bind 384-well plates were coated overnight with anti-ID antibodies specific for 10-1074. Plates were then washed, blocked, and washed again, and diluted samples, standards, and controls were added. Plates were then washed and incubated with an anti-human IgG-detection antibody, followed by washing and addition of 3,3',5,5'-Tetramethylbenzidine substrate and then stop solution. Plates were then read at 450 nm to obtain an optical density reading. Infused mAb concentration in each sample was determined based on a standard curve using a 5PL curve fit.

Several criteria were used to determine if data from an assay were acceptable and could be statistically analyzed. Any positive sample with %CV between replicates >20% was repeated. If the blank well on the plate exceeded 0.1 optical density, the plate was repeated. Standard curve EC50’s also had to be within 3 standard deviations of the historical mean EC50 as determined by optimization assays. The lowest level of quantitation was 137.15 ng/mL.

#### PK Modeling

PK was modeled using standard 2-compartment PK models parameterized in terms of clearance from the central compartment (CL), volume of the central compartment (Vc), intercompartmental distribution clearance (Q), and volume of the peripheral compartment (Vp). Rate parameters were parameterized from the PK parameters as follows:

$$\begin{array}{rr} k_{\mathrm{el}} & =\frac{\mathrm{CL}}{V_{c}}\text{,}\\ k_{12} & =\frac{Q}{V_{c}}\text{,}\\ k_{21} & =\frac{Q}{V_{p}}\text{.} \end{array}$$

Distribution and elimination half-lives were computed, respectively, as follows:

$$t_{\frac{1}{2},\alpha }=\frac{\log\left(2\right)}{\alpha }\text{,}$$

and

$$\begin{array}{r} t_{\frac{1}{2},\beta }=\frac{\log\left(2\right)}{\beta }\text{,} \end{array}$$

where α describes the distribution phase and β describes the elimination phase of the antibody. These parameters are related to the rate parameters by the following equations:

$$\alpha =\frac{-x+\sqrt{x^{2}-4y}}{2}\text{,}$$

and

$$\beta =\frac{-x-\sqrt{x^{2}-4y}}{2}\text{,}$$

where

$$x=k_{12}+k_{21}+k_{\mathrm{el}}\text{,}$$

and

$$y=k_{21}\times k_{\mathrm{el}}\text{.}$$

For each antibody, population PK models were fit using the NONMEM 7.4.2 software (ICON). NONMEM is a model analysis program that performs nonlinear mixed-effects modeling for PK analysis.

The population mean and variance were estimated for following parameters: Vc, CL, Vp, and Q. The fitted population PK model was then used to predict individual-level random effects for each parameter. A proportional error model was used.

Input dose was scaled to an amount using the weight of each animal, and administration was assumed to be bolus. The models were fit to observed concentration data above the lowest level of quantitation.

#### Statistical Tests

For each antibody, half-lives for each variant were compared using the Mann-Whitney *U* test. As this was a pilot study with a limited sample size, corrections for multiple testing were not done.

#### Animal Cohort

A total of 20 mice were infused with 10-1074 variants (4 per group) with concentrations measured at all scheduled time points after infusion. No data were missing. One sample (animal ID 14, day 2) was removed from the analysis due to an unexplained negative concentration observed. This false-negative value was confirmed by the assay laboratory.

### Ethics Statement

All mouse work was conducted according to national guidelines and was approved by the Institutional Animal Care and Use Committee of The Jackson Laboratory (AUS 15006). The Jackson Laboratory is an Assessment and Accreditation of Laboratory Animal Care-accredited and Office of Laboratory Animal Welfare–assured institution. All animal work is approved by the organization’s Institutional Animal Care and Use Committee and adheres to the standards and laws relevant to the aforementioned organizations.

The mice were euthanized at the end of the study using extended exposure (6 min) to CO_2_.

## Results

### First Round Variant Design

**Figure 1:**
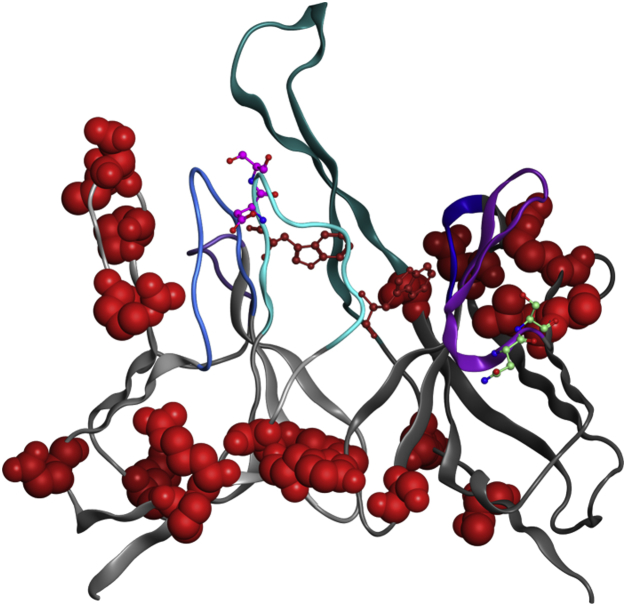
Hot spots identified in the 10-1074 parental molecule. 10-1074 hot spots were identified as described in materials and methods. Potential isomerization sites are shown in magenta, deamidation in light green, tryptophan oxidation sites in firebrick, and covariance violations in red.

While 10-1074 was isolated and cloned from sorting gp140-specific IgG memory B cells from a clade A–infected African donor as a naturally produced antibody response to HIV infection, the human antibody response is not designed to meet the needs of therapeutic manufacturing and delivery, such as large-scale production, purification, or long-term storage. A number of issues can arise during the manufacturing process including low titers, aggregation, undesirable posttranslational modifications, poor purification performance, and low pH instability during viral clearance. In addition, inherent instability of the antibodies based on colloidal and thermodynamic parameters may manifest during long-term storage as aggregation, precipitation, viscosity, and chemical instability. A series of algorithms including covariance analysis,^31^ hotspot analysis, and structural analysis were applied to the Fv region (Supplemental Table 1) to identify residues that were either potentially destabilizing, susceptible to chemical degradation, or may have led to aggregation or increased viscosity. Stability Design by Covariance Analysis uses a method known as covariance analysis to evaluate all possible residue pairs in an underlying antibody sequence database to search for specific residue positions that covary, meaning their residue properties for those sites tend to be correlated. An evaluation of the query sequence against the underlying alignment identifies its residues that violate covariance. To aid in engineering of the Fv, the Fab structure was built using the Antibody Modeler tool within the Molecular Operating Environment.^42^ This analysis (see materials and methods) resulted in 17 stability violations, one potential isomerization site, one potential deamidation site, and identification of 4 missing residues normally present in the N-terminal peptide, all of which were included in the variant design process. The first round of variant designs included the production and characterization of each site individually. Other designs included combinations of residue substitutions to build a single variant with all the sites deemed unlikely to impact function, a single variant combining the LC N-terminal residues clustered structurally, a single variant combining the LC N-terminal sequence cluster, and a series of variants containing the deleted N-terminal residues (Table 1 and Fig. 1).

Ideally, the 17 residues and their substitutions deemed likely to stabilize the Fv region of the antibody would be made into all combinations to determine the most stable form of the molecule. Doing so would require 2^∧^17 combinatorial variants, which is not practically achievable. Therefore, we devised a method to initially explore the combinatorial space by determining which of the residues potentially affected HIV neutralization, along with defining residues that increased the conformational stability of the Fab domain. Based on these data, residues we deemed the top 5 for recombination were used to construct a series of variants that were characterized for their ability to impart desirable attributes including increased thermal and chemical stability and low pH stability.

#### First-Round Characterization

The first round variants described in Table 1 were produced using transient expression in HEK293 cells and purified by protein A chromatography. Assays used for differentiation of the first round variants included neutralization, SEC to quantify monomer and HMW species after purification, and DSF to characterize stability of the modified Fab domains during thermal ramping.

The monomer content of the variants ranged from a low of 60.8% to a high of 96.3% (Supplemental Table 2). While many variants consisting of substitutions in both the LCs and HCs showed HMW of less than 10%, there were variants consisting of only substitutions in the LC that exhibited HMW levels of almost 40% by a single mutation. Two of the single LC variants, MS-204 and MS-205, which correspond to LmdV:R7P and LmdV:P9S, are near the N-terminus and exposed to the solvent. They reached aggregation levels of 34% and 28%, respectively. In contrast, modification at the LC position N+1, Y2P, showed no increase in aggregation and stabilized the Fab during thermal unfolding as discussed in the following paragraphs. Two other variants, MS-207 (H46Q) and MS-209 (I81.3S), also showed high levels of aggregation reaching 35% and 39%. Interestingly, the variant with the lowest %HMW species was MS-221, with a value of 2.5%. This variant consisted of a cluster of N-terminal residues containing the sites for MS-203, MS-204, and MS-205, supporting our contention that multiple modifications will likely produce the greatest stability. Variants with less than 10% HMW were considered for the second round combinatorial variants.

**Figure 2:**
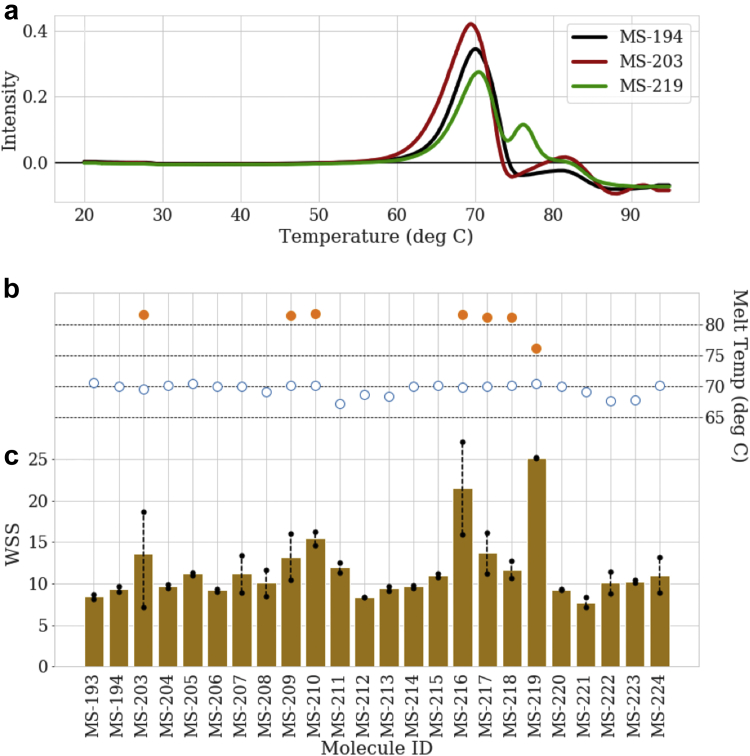
Increased thermal stability was observed for multiple round 1 variants. DSF results for select 10-1074 variants. (a) The derivative thermograms for the LS extend variant (MS-194) and 2 leading variants (MS-203, MS-219), (b) the detected melting temperatures for the CH2 (open circles) and Fab (filled circles) for all round 1 variants, and (c) the weighted shoulder score averages (bars) and replicate values (black circles with range indicated by dashed lines).

In addition to HP-SEC analysis, DSF was used to define molecules with increased thermal stability. DSF analysis of antibodies generally results in multiple transitions, with the earliest being the unfolding of the CH2 domain between 65°C and 70°C for an IgG1, followed by the Fab domain between 70°C-85°C and possibly a third transition for the CH3 domain.^43^, ^44^ The first derivative of the raw absorbance curve for the 10-1074 parental (+LS) molecule (MS-194) showed only a single transition with a Tm of 69.9°C and no clear Tm for a second transition (Fig. 2) likely due to the CH2 and Fab domains unfolding at similar temperatures. In contrast, a single mutation in the HC, HV:T108R, clearly produced a second transition with a Tm2 of 75.7°C while maintaining the original transition with a Tm1 at 70.3°C. Because only a single mutation was introduced, we posit that the second transition is due to a greater thermal stabilization of the Fab and, more specifically, the Fv domain. The presence of a second transition occurred in a subset of the variants with a distribution across both the HC and LC. The second transition in most variants though was weak as exemplified by the first derivative trace of MS-203 in Figure 2a. To provide a more quantitative analysis of the DSF data rather than only identifying the presence of Tm1 and Tm2, a metric termed the WSS was developed which accounts for the complexity and the relative temperature shift of the Fab-related peaks in the DSF curves. The results of the quantitation are shown in Figure 2b and provide for differentiation of the variants relative to their thermal stability. Based on these data, the HV:T108R mutation shows the greatest degree of stabilization as compared to the parental molecule. Other molecules with significant increases in stabilization include LmdV:Y2P (MS-203), LmdV:I81S (MS-209), LmdV:N82G (MS-210), HV:79T (MS-216), HV:R82V (MS-217), and HV:L89F (MS-218). Interestingly, the grouped combinations of variants including those with and without a second transition did not result in the observation of a second transition for those variants, see MS-221, MS-222, and MS-223. In fact, for MS-221, the Tm and WSS were lower than those of the parental molecule, suggesting a slight destabilization of the domain. Similar results were observed for the variants, with the N-terminal insertions illustrating the complex balance of amino acids defining the structural integrity of this region.

Neutralization activity was measured to ensure retention of activity of the bnAb variants. Results are shown in Supplemental Table 3 for neutralization against 6 pseudoviruses of HIV that are representative of the broader set of isolates against 10-1074. Values that are within 3-fold of the control are considered concordant by the assay validation criteria. Antibodies with more than a 3-fold increase in the IC50 or IC80 value for a particular pseudovirus were considered inactive and discarded from further consideration. As evidenced by the data, 2 variants, LmdV:H46Q (MS-207) and LmdV:P81N (MS-208), showed significantly reduced neutralization activity and were not considered for further development. The loss of neutralization activity across only a few virus isolates was confirmed by expanding the data set to an additional 6 pseudoviruses and showed that, in some cases, the neutralization activity was not concordant with the parental molecule (data not shown). Variant MS-209, which is positionally equivalent to MS-208 but a mutation to Ser rather than Asn, retained full activity across all 12 pseudoviruses tested. The combinatorial variants, MS-221 and MS-223, also showed retention of full activity, demonstrating that multiple mutations can be made in the framework region of the Fv domain and still retain full activity of the bnAbs. In contrast, the N-terminal variants demonstrated a decrease in the neutralization activity across a subset of the viruses tested.

#### Second Round Combinatorial Variant Design

The 5 residues selected for combinatorial variant analysis were MS-203 (LmdV:Y2P), MS-216 (HV:V79T), MS-217 (HV:R82V), MS-218 (HV:L89F), and MS-219 (HV:T108R). The data for selection are shown in Supplemental Tables 2 and 3. Selection criteria included an increase in conformational stability with DSF by showing a consistent Tm2, limiting the variants to MS-203, MS-209, MS-210, MS-216, MS-217, MS-218, and MS-219. Variant sites were further reduced based on HMW being less than 10%, excluding variants MS-209 and MS-210. Finally, retention of neutralization activity was required to continue inclusion of a variant. Based on these criteria, the 5 variants listed previously were included for the round 2 combinatorial variant sets. Excluding the single variants already tested, the number of combinatorial variants was 26 consisting of 10 double combinations, 10 triple combinations, 5 quadruples, and one combination consisting of all 5 amino acid modifications (Table 2).

**Table 2:** Round 2 Molecule Sets

Molecule Set^a^	Light Chain	Heavy Chain
MS-194^b^		
nMS-225	LmdV:Y2P	HV:V79T
MS-226	LmdV:Y2P	HV:R82V
MS-227	LmdV:Y2P	HV:L89F
MS-228	LmdV:Y2P	HV:T108R
MS-229		HV:V79T, HV:R82V
MS-230		HV:V79T, HV:L89F
MS-231		HV:V79T, HV:T108R
MS-232		HV:R82V, HV:L89F
MS-233		HV:R82V, HV:T108R
MS-234		HV:L89F, HV:T108R
MS-235	LmdV:Y2P	HV:V79T, HV:R82V
MS-236	LmdV:Y2P	HV:V79T, HV:L89F
MS-237	LmdV:Y2P	HV:V79T, HV:T108R
MS-238	LmdV:Y2P	HV:R82V, HV:L89F
MS-200	LmdV:Y2P	HV:R82V, HV:T108R
MS-239	LmdV:Y2P	HV:L89F, HV:T108R
MS-240		HV:V79T, HV:R82V, HV:L89F
MS-241		HV:V79T, HV:R82V, HV:T108R
MS-201		HV:V79T, HV:L89F, HV:T108R
MS-242		HV:R82V, HV:L89F, HV:T108R
MS-243	LmdV:Y2P	HV:V79T, HV:R82V, HV:L89F
MS-244	LmdV:Y2P	HV:V79T, HV:R82V, HV:T108R
MS-202	LmdV:Y2P	HV:V79T, HV:L89F, HV:T108R
MS-245	LmdV:Y2P	HV:R82V, HV:L89F, HV:T108R
MS-246		HV:V79T, HV:R82V, HV:L89F, HV:T108R
MS-247	LmdV:Y2P	HV:V79T, HV:R82V, HV:L89F, HV:T108R

a All variants including the MS-194 contain the Xtend mutation (LS) in the Fc of the heavy chain.

b MS-194 is also referred to as parental (+LS) in the manuscript.

Results of the SEC analysis for dimer and oligomer species demonstrated unique trends related to specific amino acid substitutions. No specific trends were observed for the dimer content of the variants which ranged from a low of 2.94% for MS-234 to a high of 5.61% for MS-245 (Supplemental Table 4). In contrast, the oligomer content ranged from a low of 0.36% for MS-234 to a high of 7.81% for MS-225. The variants with the lowest level of oligomer all contained the HV:T108R mutation with an average value of 0.80% ranging from 0.36% to 1.08%, while the non–HV:T108R-containing variants had an average of 3.73% oligomer ranging from 1.52% to 7.81%.

**Figure 3:**
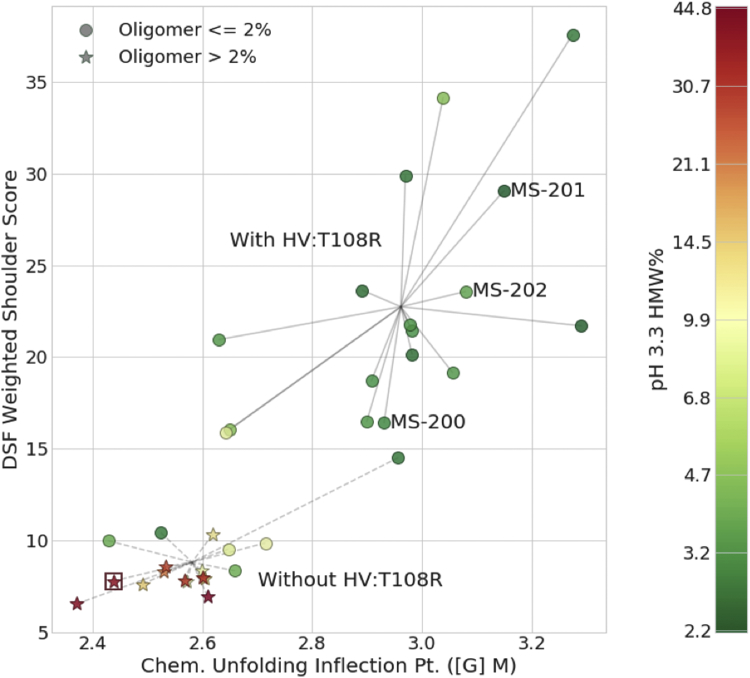
HVT108R is central to stabilizing the 10-1074 variants. Variant properties on the 3 conformational stability measures: DSF weighted shoulder score, chemical unfolding inflection point, and pH 3.3 high molecular weight (as indicated in the color bar), for all second round variants. Symbols represent high and low oligomer formation, indicated by the legend. The 2 groups formed by those variants including the HV:T108R mutation and those not including the mutation are shown by lines connecting the variants to the point representing the means of the 2 group’s properties, as labeled.

**Figure 4:**
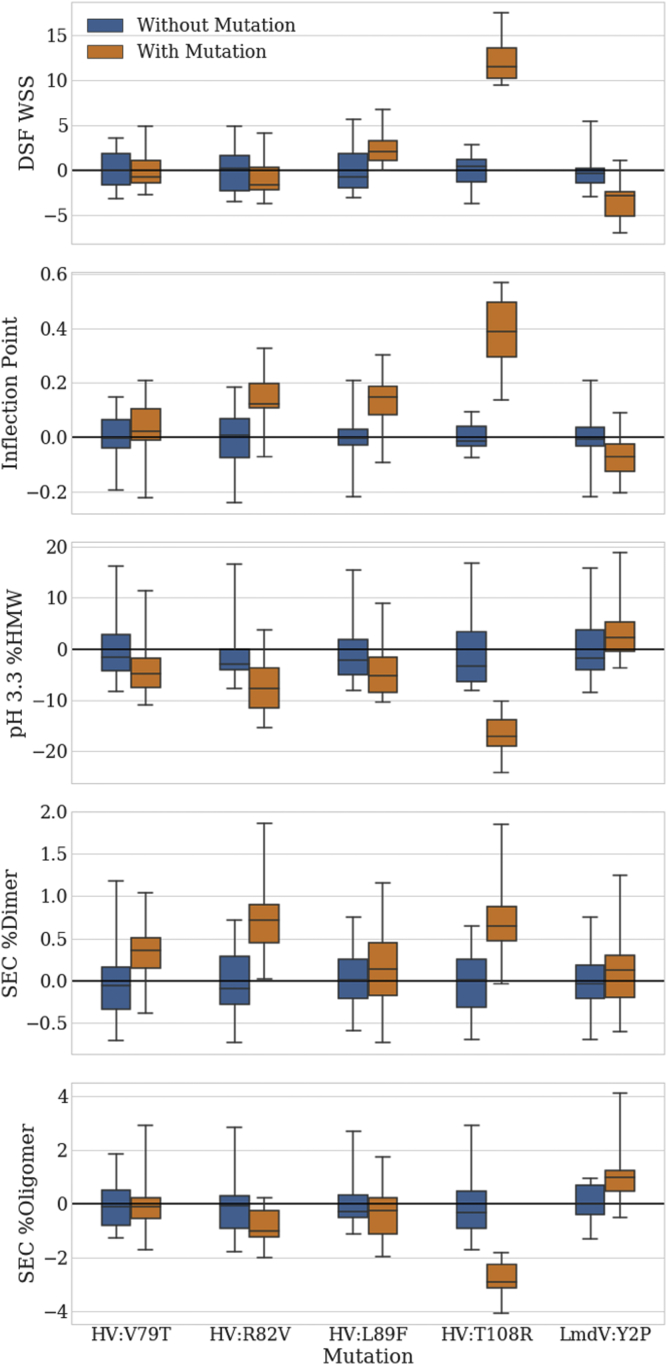
Beneficial and detrimental effects of mutations by ANOVA testing. Analysis of variance first-order effects identified for each of the 5 mutations on the 3 conformational stability measures and the 2 “detrimental” species seen in SEC (oligomer and dimer.) Each mutation’s effects are shown as the standard deviation (bars) and range (whiskers) in residuals and measured effect as an offset from the mean of the parental assay response (mean assay response without the given mutation).

Conformational stability was assessed by multiple measures including thermal ramping by DSF, chemical unfolding with Gnd-HCl, and low pH incubation followed by neutralization (Supplemental Table 5). With the DSF data, the transition temperatures alone did not clearly differentiate the thermal stability differences between the variants. For this reason, we developed the WSS, that is, an empirical aggregate quantitative measure of the difference in transition temperatures and intensities of the transitions which more clearly differentiates the thermal stability of the variants. The cumulative effects of the combinatorial modifications are plotted in Figure 3 and demonstrate the presence of 2 distinct groups, with and without HV:T108R. While this mutation appears to be a major driving force in the conformational stability, the other 4 mutations provide complementary effects. Those effects were differentiated for each of the 5 mutations on the 3 conformational stability measures and the 2 “detrimental” species seen by HP-SEC (oligomer and dimer) using analysis of variance (ANOVA) testing looking only at first-order effects. The calculated effects are plotted in Figure 4 as the effect on a property induced by each mutation relative to the corresponding molecules without the given mutation. For each effect, the residuals for the associated samples were used to show standard deviations (bars) and ranges (whiskers) associated with each effect. From these results, the beneficial and detrimental effects of each mutation can be seen. Of note again was the mutation HV:T108R which was present in all the combinatorial variant sets displaying the highest WSS and is calculated in the ANOVA as having a large, significant effect. Combinatorial variants without HV:T108R and variant MS-247 which contained all 5 mutations including HV:T108R did not display a Tm2 (Supplemental Table 4), while all the variants with HV:T108R are differentiated from the other variants by the presence of Tm2. The range of WSS for the variants with a Tm2 was from 16.01 to 29.39. The WSS for variants without a Tm2 ranged from 7.22 to 10.03, demonstrating an increase in thermal stability for some variants albeit they did not show the presence of a distinct Tm2.

Conformational stability was also assessed by chemical unfolding which interrogated the intrinsic resistance of the native state to unfolding as measured by the midpoint of the denaturation curve, referred to here as the inflection point. In contrast to the differentiation for DSF by the presence or absence of HV:T108R, most variants demonstrated an increase in the inflection point of the unfolding curve indicative of an increase in conformational stability. This is seen in the ANOVA results as notable positive inflection point effects for 3 of the 5 mutations. Only one variant, MS-225, with a double mutation of LmdV:Y2P and HV:V79T, showed a decrease in the inflection point from that of the parental molecule. The reduction in conformational stability correlated with a reduction in the WSS and Tm1. The variants with the highest inflection points all have HV:T108R, with an average value of 2.94 M compared to an average value of 2.57 M for variants without the mutation and a value of 2.44 M for the parental molecule.

Colloidal stability was measured by aggregation after low-pH incubation and by PEG solubility. For the low-pH assay, the antibody is first incubated at pH 3.3 followed by neutralization and analysis by HP-SEC. This technique allows us to interrogate both the conformational (low-pH unfolding) and colloidal stability (aggregation upon neutralization) of the molecule in a single experiment. The technique also allows us to define the expected performance of the antibody during viral inactivation. After neutralization, the aggregate level of the parental 10-1074 antibody, MS-194, increased from 8% to 40% with the majority of the aggregate being oligomer. In contrast, the second round variants showed a change in aggregate levels from −3.3% to +29.5% after neutralization with combinatorial variants containing the HV:T108R mutation showing postneutralization aggregate level changes of −3.3% to +4.5%. Other than the variant MS-228, all the molecules containing the HV:T108R mutation showed a decrease in the level of aggregation by an average of 2.2%. Solubility was also used as a measure of colloidal stability to understand the aggregation propensity at high protein concentration in the final drug product. The solubility profile of the parental molecule with increasing concentrations of PEG was mapped out, and the inflection point at 50% solubility determined to be 7.9% in PBS. This point was used to test each of the variants for either increases or decreases in solubility due to the modifications made in the framework region of the Fab. All the variants showed an increase in the solubility profile with the values ranging from 7% to 42% increase over the parental molecule indicative of an increase in colloidal stability for the variants. The ANOVA results show small decreases in aggregate levels (reported as percent high molecular weight, %HMW) for the first 3 heavy-chain mutations and a large effect for the HV:T108R mutation.

The neutralization capacity of a subset of the combinatorial variants was also examined to ensure no loss in neutralization occurred (Supplemental Table 6). A reduced set of variants were tested against a representative set of 12 pseudoviruses. Variants without a Tm2 were excluded from the testing. Of the variants that were tested, they all retained neutralization activity against the set of pseudoviruses examined.

#### Analysis of the Final Variant Set

After the biophysical analysis and neutralization assays, it was important to define the manufacturing and stability performance of the optimized antibodies before choosing a final candidate for stable pool production and scale-up. The analysis included small-scale purification and simulated viral inactivation, accelerated stability, PK analysis in human FcRn knock-in mice, and polyreactivity analysis. Because the second round combinatorial variants retained neutralization activity, the final set of variants were defined based on the biophysical attributes to provide a diversity of modification with the constraints of the 5 residues picked for round 2 combinations. Most second round variants were excluded because of lack of a Tm2, a low WSS, and a lower chemical unfolding score. The final set of variants for the in-depth analysis are shown in Table 3 and include MS-200, MS-201, and MS-202.

**Table 3:** Final Variant Set Showing Results of Viral Inactivation and Subvisible Particle Formation During Storage

Analytical Assay	MS-194		MS-200		MS-201		MS-202	
	Protein A	VI Pool	Protein A	VI Pool	Protein A	VI Pool	Protein A	VI Pool
Oligomer (%)	0.1	2.2	0.4	0.4	0.4	0.4	0.3	0.3
Dimer (%)	0.8	0.8	1.9	1.0	2.3	2.0	1.7	1.2
Total HMW (%)	0.9	3.0	2.3	1.4	2.7	2.4	2.0	1.5
	Storage time at 40°C							
	6 wk	13 wk	6 wk	13 wk	6 wk	13 wk	6 wk	13 wk
Subvisible particles ≥2 μm	12,121	39,623	2043	19,523	3027	19,321	1373	9881

### Purification and Viral Inactivation

Levels of HMW species in the intermediate product pools were measured throughout the purification process using HP-SEC (Table 3). The parent molecule (MS-194) displayed a low level of total HMW species but was susceptible to aggregation during the low-pH viral inactivation with a 22-fold increase in oligomeric species. All variant molecules demonstrated greater stability during low-pH incubation than the parent molecule and showed a reduction in dimer species after the protein A chromatography step.

### Accelerated Stability

The proteins were concentrated to 100 mg/mL because prior studies carried out in our laboratories at 1 mg/mL did not predict the long-term stability. Because the mutational analysis was focused on increasing the conformational and colloidal stability, we characterized the aggregation profile of each molecule over time by HMW formation and subvisible particle formation during a 13-week period at 40°C (Table 3). The 3 variants of 10-1074 all showed similar rates of HMW formation to the parental molecule but differentiated based on subvisible particle formation. As compared to the parental 10-1074 (MS-194), all variants showed lower amounts of subvisible particles at ≥2 μm, with MS-200 and MS-201 showing a 6-fold and 4-fold decrease, respectively, at 6 weeks while MS-202 showed a 9-fold decrease during the same period. By 13 weeks, particles in the parental molecule formulation had increased to 39,623/mL ≥2 μm, with the difference between the parental and MS-200 and MS-201 narrowing to just a 2-fold difference while the MS-202 combinatorial variant showed a 4-fold lower concentration than the parental molecule.

### Mouse PK Analysis

#### PK Models

**Figure 5:**
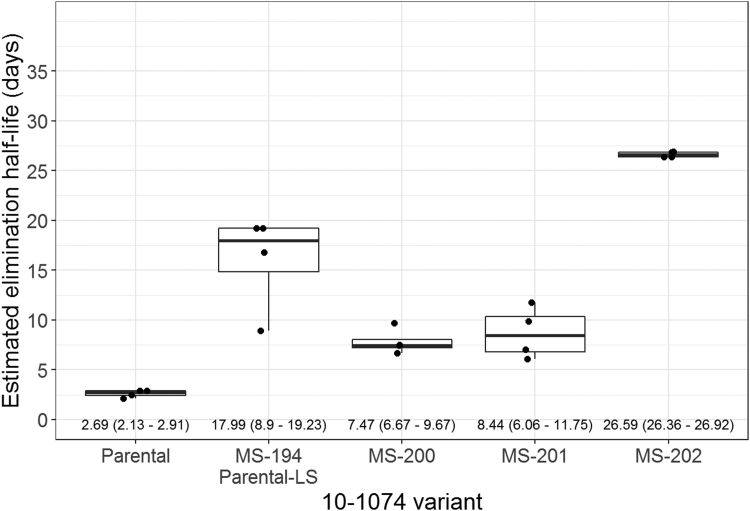
MS-202 shows the longest elimination half-life. Estimated elimination half-life (days) distributions by antibody variant group plotted as box plots with individual estimates denoted by points. The box indicates the median and interquartile range (IQR); whiskers extend to the furthest point within 1.5 times the IQR from the upper or lower quartile. Medians and ranges for each group displayed at the bottom of the plot. Individual half-lives were estimated from fitted 2-compartment population PK models for each antibody variant.

Population-level 2-compartment PK models were used to fit the data as these models have been shown to fit mAb distribution and clearance when dosed by intravenous infusion^45^ and were used to fit the clinical data for another bnAb, VRC01, that is currently in clinical trials.^46^ PK models for each group were estimated, and assessment of the PK estimates revealed differences by 10-1074 variant (Fig. 5 and Supplemental Table 7 and 8). In addition, we identified one animal (animal ID 9) in the MS-200 group that exhibited an increasing elimination rate. Based on visual inspection of increased elimination for that animal, the increased clearance rate was attributed to a potential anti-drug antibody response, and a second PK model for MS-200 was fit excluding that animal’s data after day 10. The model-fitted curves and elimination half-lives were stable for the remaining animals receiving MS-200, but fitted values of individual PK parameters did exhibit a slight change (Supplemental Table 7). Within group, PK parameters displayed stability between the animals. Variation across analytes for V_P_ and Q indicated potential varying kinetics in the distributional phase for the animals.

#### Elimination Half-lives

The MS-202 variant had the longest elimination half-life for the 10-1074 antibody with a median elimination half-life of 26.59 (range: 26.36-26.92) days. The parental-LS (MS-194) had the second longest elimination half-life (median: 17.99; range: 8.9-19.23 days) (Fig. 5). Estimated half-life medians (range) from groups MS-201, MS-200, and the parental (non-LS) molecule were 8.44 (6.06-11.75), 7.47 (6.67-9.67), and 2.69 (2.13-2.91) days, respectively. Significant differences were detected between the elimination half-lives of each of the variant strains and the parental (non-LS) strain (all *p* = 0.010; Supplemental Table 9). In addition, the elimination half-life for MS-202 was significantly different from the elimination half-life for MS-200, MS-201, and MS-194 (all *p* = 0.021). The LS-mutant (MS-194) elimination half-life was also significantly different than the elimination half-life for MS-200 (*p* = 0.043).

### Polyreactivity

**Figure 6:**
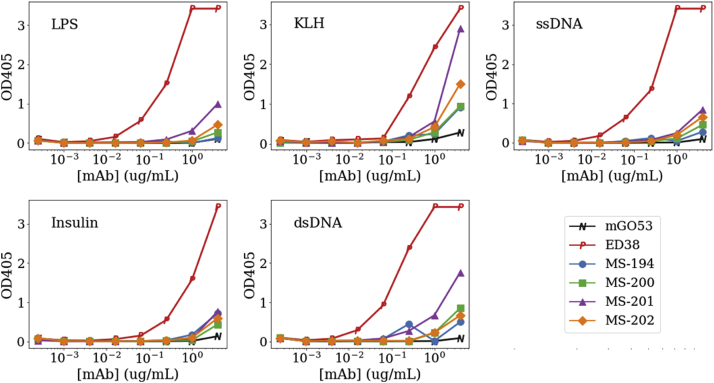
An increased polyreactivity was associated with the MS-201 variant. Polyreactivity for the top 3 10-1074 second round variants along with the LS extend parent (MS-194) and a positive and negative control (ED38 and mGO53, respectively).

High degrees of somatic hypermutation as occurs for the bnAbs is known to significantly increase the nonspecific polyreactivity of antibodies.^40^ Although the starting antibody was known to have low polyreactivity, and the mutations to fix the covariance violations were conservative residue replacements, we still felt it important to characterize the possible changes in the polyreactivity of the variants. Polyreactivity was examined against a defined set of antigens including single-stranded DNA, double-stranded DNA, lipopolysaccharide, insulin, and keyhole lymphocyte hemocyanin (Fig. 6). ED38 protein was used as a positive control and showed binding to all reagents while 10-1074-LS (MS-194), MS-200, and MS-202 showed minimal binding by the assay. In contrast, MS-201, which showed little or no binding to SS-DNA and insulin, did show a minimal binding to lipopolysacharide with additional increase to double-stranded DNA and an apparent significant increase with Keyhole limpet hemocyanin, suggesting some level of polyreactivity that may manifest itself after injection.

## Discussion

Over the past 10 years, a number of bnAbs have been isolated^47^ and shown to prevent acquisition of HIV in macaques after multiple challenges with simian/human immunodeficiency viruses.^30^, ^48^, ^49^, ^50^ A small number of these bnAbs are now in clinical trials for demonstrating their use in preexposure prophylaxis as either an alternative or adjunct to pre-exposure prophylaxis therapy^14^, ^15^, ^16^, ^29^, ^30^, ^41^ because of their alternative mode of action and long half-life. The eventual success of these molecules in developing countries such as sub-Saharan Africa will be dependent not only on their efficacy but also on their ability to be produced at low cost and to minimize supply chain problems and their ease of delivery for the patient. Based on models developed by Garcia and Vandiver,^18^ lower cost biologics are achievable with the development of lean and efficient bioprocessing facilities through footprint reduction and adoption of disposable and continuous manufacturing technologies. A major limiting constraint on plant performance in these facilities, and ultimately cost, is the stability of the neutralized viral inactivated pool which is directly affected by the conformational stability of the molecule. In addition to cost, another difficulty for getting biologics into sub-Saharan Africa is stability of the molecule outside the cold chain. While adequate cold chain may exist in major cities, outside the cities, the cold chain and electric grid are less reliable. Only 50% of target health-care posts are on-grid with at least 8 h of electricity per day.^51^ Product stability at room temperature is then a major concern. With average daily temperatures up to 30°C,^52^ it may be possible to obtain enough time outside the cold chain to avoid or significantly reduce refrigeration requirements at rural health posts by designing more stable therapeutics. Similar to viral inactivation, protein aggregation, a major degradant during room temperature storage, is related to conformational stability. This would suggest that it is reasonable to expect that increasing conformational stability, thereby lowering protein aggregation, will lead to increased storage stability necessary for delivery of a product in more remote regions of sub-Saharan Africa.

Multiple publications have shown that modification of residues within the Fv region can increase the conformational stability of the Fab^20^, ^21^, ^22^, ^24^, ^53^, ^54^ with greater increases associated with multiple modifications. Here we specifically targeted residues in the framework regions 1-4 using a purely computational approach based on the method of Gunasekaran et al.^31^ to determine which could potentially destabilize the domain. Residues of the CDR loops 1-3 were considered integral to the binding and neutralization activity of the bnAb and were not modified. Prior work by Klein et al.^8^ showed that somatic mutations of the framework region, which we targeted, were required for the broad and potent HIV-1 neutralization and that mutations at these sites back to germline lead to a loss in neutralization activity. In our work, we identified potentially destabilizing residues based on covariance violations analysis by comparing with publicly accessible databases. By doing this, not all somatically hypermutated residues were targeted, and in fact, many of the residues mutated to germline by Klein et al.^8^ did not demonstrate significant covariance violations and were not mutated. In addition, we did not always mutate residues back to germline but rather back to residues in line with covariance based on our database.

We identified 17 potentially destabilizing residues in the framework region. Ideally, we would test all combinations of the residue replacements resulting in 2^∧^17 combinatorial variants. Because it is unreasonable to produce and characterize this number of molecules, we devised a strategy based on first determining which residues, as single variants, were important for neutralization activity. Surprisingly, only 2 of the residues, LmdV:H46Q and LmdV:P81N, showed a loss in activity, and this was only for a limited number of HIV isolates with full neutralization activity for other isolates with these variants. LmdV:H46Q is at the bottom of the Fv but points toward the variable light core and may partially disrupt packing. LmdV:P81N is on the outside edge of the framework 3 loop and interacts with the gp120 glycan. Mutation from a Pro to an Asn may disrupt the interaction either directly or indirectly through perturbation of the structure leading to a decrease in binding. Together, this not only demonstrates the high degree of specificity of interactions with different HIV isolates but also the plasticity of the interactions of residues within the framework. We also found that as long as a modified residue retained neutralization activity, combinations of these residues retained activity, demonstrating the independent nature of the interactions with the HIV.

To explore the conformational space of the combinatorial variants, we used DSF to characterize the conformational stability during thermal unfolding, Gnd-HCl denaturation to characterize conformation and side chain packing stability of the core residues during chemical unfolding, and low-pH stability to explore the conformational and colloidal stability during low-pH stress. Combining these 3 techniques allowed us to gain a more global view of different energetics leading to the stability of the Fab domain. Based on these studies, the central role of the HV:T108R mutation stabilizing the HV-CDR3 became apparent. The parental molecule showed only a single melting transition likely comprised of both the CH2 and Fab domains based on differential scanning calorimetry thermal unfolding profiles of mAbs.^43^ Of the single site mutations, the HV:T108R mutation showed the highest WSS and the most distinct Tm2 transition. Similar observations were made for the combinatorial variants that contained the HV:T108R mutation compared with the parental molecule and variants without the mutation (Fig. 3). This central role was confirmed through studies using chemical unfolding with Gnd-HCl in which the combinatorial variants with the HV:T108R showed an average increase in guanidine concentration of 0.5M for the inflection point. The stabilization carried through to resistance by low pH unfolding in which the single variant showed a 4% increase in HMW formation while the parental molecule showed a 40% HMW formation after neutralization. In the context of combinatorial variants, those containing the HV:T108R mutation all demonstrated resistance to aggregation upon neutralization from the low-pH incubation.

**Figure 7:**
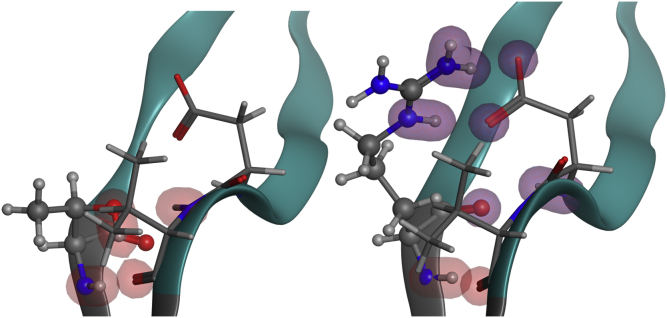
HV:T108R mutation forms multiple ionic interactions to stabilize CDR3. Contact analysis of parental 10-1074 with HV:T108 (left) and HV:R108 (right). With HV:T108, 3 hydrogen bonds are formed with HV:V138. With the HV:R108 variant, there are 4 ionic bonds and 4 hydrogen bonds with HV:D137 and one hydrogen bond with HV:V138. Ionic interactions are shown by the purple contact shells, and the hydrogen bonds are shown by the red contact shells.

In the parental form of 10-1074, the antibody contains Thr at the ASN position HV:108, which forms hydrogen bonds the backbone of HV:V138 (Fig. 7) with a calculated energy of −9.3 kcal/mol. The presence of HV:T108 in 10-1074, however, produces a high number of covariance violations in the IGHV4 subgroup. A positional frequency analysis of this position indicates that within this subgroup, the site is Arg at a frequency of 82%. An evaluation of the germlines closest to 10-1074 also indicates that Arg is preferred at this position and that Asp is preferred at position HV:137, which is the residue found at HV:137 in 10-1074. A mutation of HV:T108R removes the covariance violation. Examination of the Fv structure in the region of HV:T108 clearly demonstrates the stabilizing role in the structure. HV:T108 is located at the base of the extended HC-CDR3 loop forming a hydrogen bond contact between the hydroxyl oxygen and the HV:V138 amide hydrogen of the opposing β-strand on the base of the other side of the HC-CDR3 loop. The calculated energy of this H-bond is −9.3 kcal/mol. By mutating the HC:T108 to an Arg residue (structure modeling performed using the antibody modeler tool within the Molecular Operating Environment^42^), the guanidino group of the Arg can now interact with the carboxyl group of HV:D137 on the opposing β-strand at the C-terminus of the CDR3 loop resulting in a calculated −36.3 kcal/mol through ionic interactions with HV:D137 and −4.9 kcal/mol through H-bond interactions with HV:V138, effectively stabilizing the CDR3 loop. We believe it is this stabilization that is manifested in the thermal, chemical, and low pH unfolding of the molecule. We hypothesize that during low-pH incubation of the parental molecule, the carboxylate of HV:D137 is protonated and destabilizes the HC-CDR3 loop such that when the antibody is neutralized, the hydrophobic and nonpolar residues of the loop interact with those of other molecules forming dimers and oligomers. The salt bridge between the guanidine group of HV:R108 with the carboxylate of HV:D137 prevents protonation of the carboxylate such that the Fv remains folded at low pH. An evaluation of existing antibody structures was performed using the SAbDab^55^ database to list human antibodies with Kabat-defined HC-CDR3s with a crystal structure resolution better than 3.0 Å. This analysis indicates that the presence of HV:R108-HV:D137 occurs 290 times in the 664 structures evaluated, illustrating a high occurrence of this ionic interaction.

In addition to the obvious importance of HV:T108R in stabilizing the Fv region, other amino acids together were necessary for further stabilization. Initially, we picked the mutations LmdV:Y2P, HV:V79T, HV:R82V, and HV:L89F based on stabilization observed through thermal analysis and proceeded with the combinatorial variants. The double combinatorials with the LmdV:Y2P appeared to destabilize the molecule through a decrease in the value of the WSS from the single variants to the combinatorials. The WSS analysis was developed after we proceeded to round 2 combinatorials making the analysis of the singles retrospective. The destabilizing effect carried through to the triple combinatorials as well, with the addition of the LmdV:Y2P only modestly affecting the WSS and the chemical unfolding inflection point value while causing a significant increase in the %HMW after low-pH hold and neutralization. For instance, the HV:V79T/HV:L89F had an HMW value of 12%, while the presence of LmdV:Y2P increased the value to 22%. The 10-1074 parental bnAb has a 3 amino acid deletion between residues 4-6, 8, and 10 at the N-terminus of the LC causing a dramatic change in the position of the N-terminus from other antibodies without the deletions.^56^ An evaluation of the LC N-terminal strand via LowMode molecular dynamics (MOE) was performed with both LmdV:Y2 and LmdV:P2 versions, resulting in no obvious stabilizing interactions for the LmdV:Y2 form. However, the presence of a hydroxyl group on the tyrosine would allow for hydrogen bonding interactions after some loosening of the structure during low pH stress and that could stabilize the LmdV:Y2 form during pH neutralization. Other double combinatorials demonstrated increased stability with those containing the LmdV:T108R showing the most improvement across all assays with the double combinatorial HV:L89F/HV:T108R performing the best. Structurally, HV:L89 is in a hydrophobic pocket between HV-CDR1 and HV-CDR2 containing the indole ring of HV:W41 and side chain of HV:I58. Substituting Phe for Leu at position 89 would allow for pi-pi ring stacking interactions after some rearrangement of the residues leading to increased stability with this carrying into the triple and quadruple variants.

The effect of the optimization process was clearly defined by molecules with increased conformational stability, but other factors were necessary to consider to define a molecule with the needed manufacturability and stability profiles. Three molecules were picked with varying degrees of conformational stability to test in our models. While scale-down studies of the purification process showed no obvious problems with purification, they did validate the use of the low-pH model to predict aggregation during the viral inactivation step. The results of the stability studies at 40°C were unexpected in demonstrating that while the rates of HMW formation were unchanged, the formation of subvisible particles was greatly reduced, with the quadruple combinatorial variant, MS-202, showing the greatest reduction during storage. This itself provides an advantage toward achieving some room temperature stability in sub-Saharan Africa because of the expected variation in temperatures across the region.^57^ In addition, this would provide for greater time outside the cold chain as is expected in rural areas without a consistent source of electricity. Recent approvals of mAb-based biologics such as Amjevita® (Amgen) and Dupixent® (Regeneron) have shown that the agencies are willing to allow for room temperature stability albeit an upper temperature limit of 25°C for 14 days is placed on the product. While this is reasonable, it would be difficult to verify, necessitating the need for stability above the stated temperatures or the length of time.

The modifications of these antibodies were based on those commonly observed in mAb databases based on the structural identity at each residue and not necessarily based on germline residues. It was not necessarily surprising then that one combinatorial variant, MS-201, showed an increased signal in the polyreactivity assay. Studies by Wardemann et al.^40^ have demonstrated that during the somatic mutation process of antibody maturation, numerous mutations from germline are introduced that result in increased polyreactivity. A major difference between MS-201 and the other 2 combinatorial variants was the presence of HV:L89F, which by itself, would not be expected to increase polyreactivity as it is buried within the interior of the Fv. It seems likely then that the increased polyreactivity was the result of minor structural rearrangements on the exterior surface of the bnAb possibly exposing the HV:F89 to the solvent. Tissue cross-reactivity studies against a variety of human tissue samples (data not shown) were negative for any of the bnAbs other than nonspecific cytoplasm-binding ones. Because 10-1074 is already in the clinic and negative for polyreactivity, the increase for MS-201 was enough to exclude it for being chosen as the final optimized variant.

The elimination half-life of 10-1074 in humans is 24 days^16^ and not optimal for maintaining adequate trough levels in preexposure prophylaxis if injections are delivered every 3 months. Therefore, the Xencor Xtend^58^ Fc mutations were introduced to increase half-life and maintain adequate trough levels with dosing every 3 months. The final dose is dependent on a low-enough COGs allowing for treatment in the sub-Saharan Africa region. Testing of the variants in a Tg276 mouse model,^59^, ^60^ which has been shown to correlate to half-life in humans, interestingly demonstrated a possible effect of mutations in the Fv region on the half-life of the molecule. All variants with the Xtend mutation had a longer elimination half-life than the parental molecule without the Xtend mutation. The MS-202 variant showed a half-life that was greater than that of the other 2 variants leading us to pick the MS-202 variant as the final optimized form. The reason for the differences in half-life between the variants is unclear and may be related to variability in the assay due to the minimal purification of the molecules. We state this as comparison of the MS-202 variant with the Parental-LS in the same model shows no difference between the 2 molecules when both are produced using stable cell lines and GMP manufacturing practices even though they show a statistically significant difference here. Because of the inherent variability in animal studies, statistically significant differences between variant elimination half-lives should be interpreted cautiously when applied to human studies. However, studies by Wang et al.^61^ of mAbs with the same wild-type human Fc sequences but different Fab domains were shown to bind FcRn with considerable differences in both the binding at acidic pH and the dissociation at neutral pH, suggesting that the Fab domain may also have an impact on FcRn interaction. Therefore, we cannot rule out a true difference between the variants although additional studies in the *in vivo* model used here would be required to sort this out.

## Conclusions

Bringing biologics such as bnAbs into developing countries is a daunting task because of multiple issues including logistics, COGs, and cold chain distribution. While cold chain distribution lanes are in place for vaccines, they are often filled to capacity such that bringing in bnAbs through these established systems may initially be difficult. Rural areas outside of the major cities will prove to be problematic because of the lack of a stable electric grid and transportation of the drug product within a cold chain. Designing the molecule to better deal with these problems such as time outside the cold chain and high temperature stress will go a long way toward helping solve many of the problems we expect to encounter. COGs is another issue that will be just as difficult but manageable with the manufacturing costs decreasing over time. Many areas of manufacturing are ripe for cost reduction from the bioreactor productivity to downstream processing and size and flexibility of the manufacturing plant. These improvements, together with dose reductions due to increased half-life, will help lead to introduction of these products to the market in low- and middle-income countries to reduce, and eventually eliminate, the global spread of HIV.
