# Supplementary material for: Framework Mutations of the 10-1074 bnAb Increase Conformational Stability, Manufacturability, and Stability While Preserving Full Neutralization Activity
Source: J Pharm Sci. 2020 Jan;109(1):233–46. doi: 10.1016/j.xphs.2019.07.009 (PMC6941225; doi:10.1016/j.xphs.2019.07.009)
Supplement: Supplemental Tables [file mmc1.docx]

**Supplemental Table 1:** Heavy and light chain Fv residue numbering. Numbering is based on the structural alignment method of Honegger and PluÈckthun (J. Mol. Biol., 2001, 309: ﻿657-670).

| **10-1074 Light Chain** | | | | | | | | |
| --- | --- | --- | --- | --- | --- | --- | --- | --- |
| **Residue** | **Mat. Linear #** | **ASN #** | **Residue** | **Mat. Linear #** | **ASN #** | **Residue** | **Mat. Linear #** | **ASN #** |
| S | 1 | LmdV:1 | H | 33 | LmdV:46 | R | 68 | LmdV:88 |
| Y | 2 | LmdV:2 | R | 34 | LmdV:47 | A | 69 | LmdV:89 |
| V | 3 | LmdV:3 | P | 35 | LmdV:48 | T | 70 | LmdV:90 |
| - | 3.1 | LmdV:4 | G | 36 | LmdV:49 | L | 71 | LmdV:91 |
| - | 3.2 | LmdV:5 | Q | 37 | LmdV:50 | T | 72 | LmdV:92 |
| - | 3.3 | LmdV:6 | A | 38 | LmdV:51 | I | 73 | LmdV:93 |
| R | 4 | LmdV:7 | P | 39 | LmdV:52 | S | 74 | LmdV:94 |
| - | 4.1 | LmdV:8 | I | 40 | LmdV:53 | G | 75 | LmdV:95 |
| P | 5 | LmdV:9 | L | 41 | LmdV:54 | V | 76 | LmdV:96 |
| - | 5.1 | LmdV:10 | L | 42 | LmdV:55 | E | 77 | LmdV:97 |
| L | 6 | LmdV:11 | I | 43 | LmdV:56 | A | 78 | LmdV:98 |
| S | 7 | LmdV:12 | Y | 44 | LmdV:57 | G | 79 | LmdV:99 |
| V | 8 | LmdV:13 | N | 45 | LmdV:58 | D | 80 | LmdV:100 |
| A | 9 | LmdV:14 | - | 45.1 | LmdV:59 | E | 81 | LmdV:101 |
| L | 10 | LmdV:15 | - | 45.2 | LmdV:60 | A | 82 | LmdV:102 |
| G | 11 | LmdV:16 | - | 45.3 | LmdV:61 | D | 83 | LmdV:103 |
| E | 12 | LmdV:17 | - | 45.4 | LmdV:62 | Y | 84 | LmdV:104 |
| T | 13 | LmdV:18 | - | 45.5 | LmdV:63 | Y | 85 | LmdV:105 |
| A | 14 | LmdV:19 | - | 45.6 | LmdV:64 | C | 86 | LmdV:106 |
| R | 15 | LmdV:20 | - | 45.7 | LmdV:65 | H | 87 | LmdV:107 |
| I | 16 | LmdV:21 | - | 45.8 | LmdV:66 | M | 88 | LmdV:108 |
| S | 17 | LmdV:22 | N | 46 | LmdV:67 | W | 89 | LmdV:109 |
| C | 18 | LmdV:23 | Q | 47 | LmdV:68 | D | 90 | LmdV:110 |
| G | 19 | LmdV:24 | D | 48 | LmdV:69 | S | 91 | LmdV:111 |
| R | 20 | LmdV:25 | R | 49 | LmdV:70 | R | 92 | LmdV:112 |
| Q | 21 | LmdV:26 | P | 50 | LmdV:71 | - | 92.1 | LmdV:113 |
| - | 21.1 | LmdV:27 | S | 51 | LmdV:72 | - | 92.2 | LmdV:114 |
| - | 21.2 | LmdV:28 | G | 52 | LmdV:73 | - | 92.3 | LmdV:115 |
| - | 21.3 | LmdV:29 | I | 53 | LmdV:74 | - | 92.4 | LmdV:116 |
| A | 22 | LmdV:30 | P | 54 | LmdV:75 | - | 92.5 | LmdV:117 |
| L | 23 | LmdV:31 | E | 55 | LmdV:76 | - | 92.6 | LmdV:118 |
| G | 24 | LmdV:32 | R | 56 | LmdV:77 | - | 92.7 | LmdV:119 |
| S | 25 | LmdV:33 | F | 57 | LmdV:78 | - | 92.8 | LmdV:120 |
| - | 25.1 | LmdV:34 | S | 58 | LmdV:79 | - | 92.9 | LmdV:121 |
| - | 25.2 | LmdV:35 | G | 59 | LmdV:80 | - | 92.1 | LmdV:122 |
| - | 25.3 | LmdV:36 | T | 60 | LmdV:81 | - | 92.11 | LmdV:123 |
| - | 25.4 | LmdV:37 | P | 61 | LmdV:81.1 | - | 92.12 | LmdV:124 |
| - | 25.5 | LmdV:38 | D | 62 | LmdV:81.2 | - | 92.13 | LmdV:125 |
| R | 26 | LmdV:39 | I | 63 | LmdV:81.3 | - | 92.14 | LmdV:126 |
| A | 27 | LmdV:40 | N | 64 | LmdV:82 | - | 92.15 | LmdV:127 |
| V | 28 | LmdV:41 | F | 65 | LmdV:83 | - | 92.16 | LmdV:128 |
| Q | 29 | LmdV:42 | G | 66 | LmdV:84 | - | 92.17 | LmdV:129 |
| W | 30 | LmdV:43 | - | 66.1 | LmdV:85 | - | 92.18 | LmdV:130 |
| Y | 31 | LmdV:44 | - | 66.2 | LmdV:86 | - | 92.19 | LmdV:131 |
| Q | 32 | LmdV:45 | T | 67 | LmdV:87 | - | 92.2 | LmdV:132 |
|  |  |  |  |  |  |  |  |  |
| **10-1074_LC** |  |  |  |  |  |  |  |  |
| **Residue** | **Mat. Linear #** | **ASN #** |  |  |  |  |  |  |
| G | 94 | LmdV:134 |  |  |  |  |  |  |
| F | 95 | LmdV:135 |  |  |  |  |  |  |
| S | 96 | LmdV:136 |  |  |  |  |  |  |
| W | 97 | LmdV:137 |  |  |  |  |  |  |
| S | 98 | LmdV:138 |  |  |  |  |  |  |
| F | 99 | LmdV:139 |  |  |  |  |  |  |
| G | 100 | LmdV:140 |  |  |  |  |  |  |
| G | 101 | LmdV:141 |  |  |  |  |  |  |
| A | 102 | LmdV:142 |  |  |  |  |  |  |
| T | 103 | LmdV:143 |  |  |  |  |  |  |
| R | 104 | LmdV:144 |  |  |  |  |  |  |
| L | 105 | LmdV:145 |  |  |  |  |  |  |
| T | 106 | LmdV:146 |  |  |  |  |  |  |
| V | 107 | LmdV:147 |  |  |  |  |  |  |
| L | 108 | LmdV:148 |  |  |  |  |  |  |
| G | 109 | LmdV:149 |  |  |  |  |  |  |

| **10-1074 Heavy Chain** | | | | | | | | |
| --- | --- | --- | --- | --- | --- | --- | --- | --- |
| **Residue** | **Mat. Linear #** | **ASN #** | **Residue** | **Mat. Linear #** | **ASN #** | **Residue** | **Mat. Linear #** | **ASN #** |
| Q | 1 | HV:1 | G | 44 | HV:51 | T | 90 | HV:101 |
| V | 2 | HV:2 | L | 45 | HV:52 | A | 91 | HV:102 |
| Q | 3 | HV:3 | E | 46 | HV:53 | V | 92 | HV:103 |
| L | 4 | HV:4 | W | 47 | HV:54 | Y | 93 | HV:104 |
| Q | 5 | HV:5 | I | 48 | HV:55 | Y | 94 | HV:105 |
| E | 6 | HV:6 | G | 49 | HV:56 | C | 95 | HV:106 |
| S | 7 | HV:7 | Y | 50 | HV:57 | A | 96 | HV:107 |
| - | 7.1 | HV:8 | I | 51 | HV:58 | T | 97 | HV:108 |
| G | 8 | HV:9 | S | 52 | HV:59 | A | 98 | HV:109 |
| P | 9 | HV:10 | D | 53 | HV:60 | R | 99 | HV:110 |
| G | 10 | HV:11 | - | 53.1 | HV:61 | R | 100 | HV:111 |
| L | 11 | HV:12 | - | 53.2 | HV:62 | G | 101 | HV:112 |
| V | 12 | HV:13 | - | 53.3 | HV:63 | Q | 102 | HV:113 |
| K | 13 | HV:14 | - | 53.4 | HV:64 | R | 103 | HV:114 |
| P | 14 | HV:15 | R | 54 | HV:65 | I | 104 | HV:115 |
| S | 15 | HV:16 | E | 55 | HV:66 | Y | 105 | HV:116 |
| E | 16 | HV:17 | S | 56 | HV:67 | G | 106 | HV:117 |
| T | 17 | HV:18 | A | 57 | HV:68 | V | 107 | HV:118 |
| L | 18 | HV:19 | T | 58 | HV:69 | V | 108 | HV:119 |
| S | 19 | HV:20 | Y | 59 | HV:70 | - | 108.1 | HV:120 |
| V | 20 | HV:21 | N | 60 | HV:71 | - | 108.2 | HV:121 |
| T | 21 | HV:22 | P | 61 | HV:72 | - | 108.3 | HV:122 |
| C | 22 | HV:23 | S | 62 | HV:73 | - | 108.4 | HV:123 |
| S | 23 | HV:24 | L | 63 | HV:74 | - | 108.5 | HV:124 |
| V | 24 | HV:25 | N | 64 | HV:75 | - | 108.6 | HV:125 |
| S | 25 | HV:26 | S | 65 | HV:76 | S | 109 | HV:126 |
| G | 26 | HV:27 | R | 66 | HV:77 | F | 110 | HV:127 |
| - | 26.1 | HV:28 | V | 67 | HV:78 | G | 111 | HV:128 |
| D | 27 | HV:29 | V | 68 | HV:79 | E | 112 | HV:129 |
| S | 28 | HV:30 | I | 69 | HV:80 | F | 113 | HV:130 |
| M | 29 | HV:31 | S | 70 | HV:81 | F | 114 | HV:131 |
| N | 30 | HV:32 | R | 71 | HV:82 | Y | 115 | HV:132 |
| N | 31 | HV:33 | D | 72 | HV:83 | Y | 116 | HV:133 |
| - | 31.1 | HV:34 | T | 73 | HV:84 | Y | 117 | HV:134 |
| - | 31.2 | HV:35 | S | 74 | HV:85 | S | 118 | HV:135 |
| - | 31.3 | HV:36 | K | 75 | HV:86 | M | 119 | HV:136 |
| - | 31.4 | HV:37 | N | 76 | HV:87 | D | 120 | HV:137 |
| - | 31.5 | HV:38 | Q | 77 | HV:88 | V | 121 | HV:138 |
| Y | 32 | HV:39 | L | 78 | HV:89 | W | 122 | HV:139 |
| Y | 33 | HV:40 | S | 79 | HV:90 | G | 123 | HV:140 |
| W | 34 | HV:41 | L | 80 | HV:91 | K | 124 | HV:141 |
| T | 35 | HV:42 | K | 81 | HV:92 | G | 125 | HV:142 |
| W | 36 | HV:43 | L | 82 | HV:93 | T | 126 | HV:143 |
| I | 37 | HV:44 | N | 83 | HV:94 | T | 127 | HV:144 |
| R | 38 | HV:45 | S | 84 | HV:95 | V | 128 | HV:145 |
| Q | 39 | HV:46 | V | 85 | HV:96 | T | 129 | HV:146 |
| S | 40 | HV:47 | T | 86 | HV:97 | V | 130 | HV:147 |
| P | 41 | HV:48 | P | 87 | HV:98 | S | 131 | HV:148 |
| G | 42 | HV:49 | A | 88 | HV:99 | S | 132 | HV:149 |

**Supplemental Table 2:** Analytical characterization of round 1 variants.

| Molecule Set | HP-SEC  (% Monomer) | HP-SEC  (% HMW) | DSF Tm1, °C | DSF Tm2, °C |
| --- | --- | --- | --- | --- |
| MS-194 | 91.52 | 8.48 | 69.9 ± 0.14 |  |
| MS-203 | 95.24 | 4.76 | 69.3 ± 0.00 | 81.35 ± 0.35 |
| MS-204 | 65.98 | 34.02 | 70.0 ± 0.14 |  |
| MS-205 | 71.66 | 28.34 | 70.2 ± 0.07 |  |
| MS-206 | 91.35 | 8.65 | 70.0 ± 0.00 |  |
| MS-207 | 65.30 | 34.70 | 69.9 ± 0.07 | 80.80 |
| MS-208 | 88.68 | 11.32 | 68.9 ± 0.14 | 81.30 |
| MS-209 | 60.79 | 39.21 | 70.1 ± 0.07 | 80.70 ± 0.85 |
| MS-210 | 88.24 | 11.76 | 70.2 ± 0.14 | 81.50 ± 0.28 |
| MS-211 | 83.11 | 16.89 | 67.0 ± 0.14 |  |
| MS-212 | 82.78 | 17.22 | 68.6 ± 0.07 |  |
| MS-213 | 90.48 | 9.52 | 68.2 ± 0.07 |  |
| MS-214 | 94.00 | 6.00 | 69.9 ± 0.00 |  |
| MS-215 | 92.88 | 7.12 | 70.1 ± 0.07 |  |
| MS-216 | 94.67 | 5.33 | 69.9 ±0.07 | 80.95 ± 1.20 |
| MS-217 | 94.21 | 5.79 | 69.9 ± 0.07 | 80.50 ± 0.57 |
| MS-218 | 96.33 | 3.67 | 70.1 ± 0.07 | 81.10 ± 0.28 |
| MS-219 | 91.93 | 8.07 | 70.3 ± 0.00 | 75.70 ± 0.42 |
| MS-220 | 94.56 | 5.44 | 69.9 ± 0.00 |  |
| MS-224 |  |  | 70.1 ± 0.21 | 80.80 |
| MS-221 | 97.54 | 2.46 | 68.8 ± 0.18 |  |
| MS-222 | 89.79 | 10.21 | 67.4 ± 0.11 |  |
| MS-223 | 84.14 | 15.86 | 67.5 ± 0.01 |  |
| MS-257 | 85.00 | 15.00 | 68.5 ± 0.09 |  |
| MS-258 | 85.79 | 14.21 | 68.6 ± 0.03 |  |
| MS-259 | 79.79 | 20.21 | 68.1 ± 0.03 |  |
| MS-260 | 84.66 | 15.34 | 68.5 ± 0.10 |  |

**Supplemental Table 3:** Round 1 molecule sets and neutralization analysis against 10-1074 sensitive virus panel in TZM.bl cells. Loss of potency are values > 3-fold of control value. Assay Set up: mAbs tested at primary concentration of 25 ug/ml and titrated 5-fold 7x (duplicate wells). Due to known assay variability, a new control was run with each set of samples.

|  | **Du156.12** | | **WITO4160.33** | | **CNE17** | | **CNE30** | | **CAAN5342.A2** | | **Du172.17** | |
| --- | --- | --- | --- | --- | --- | --- | --- | --- | --- | --- | --- | --- |
| **Molecule Set** | **IC50** | **IC80** | **IC50** | **IC80** | **IC50** | **IC80** | **IC50** | **IC80** | **IC50** | **IC80** | **IC50** | **IC80** |
| Control | 0.010 | 0.030 | 0.168 | 1.173 | 1.201 | 6.067 | 0.164 | 0.605 | 0.009 | 0.029 | 0.110 | 0.391 |
| MS-203 | 0.008 | 0.025 | 0.205 | 0.979 | 1.291 | 4.519 | 0.193 | 0.546 | 0.009 | 0.024 | 0.084 | 0.295 |
| MS-204 | 0.014 | 0.040 | 0.236 | 1.664 | 1.211 | 5.895 | 0.270 | 0.902 | 0.017 | 0.048 | 0.092 | 0.334 |
| MS-205 | 0.012 | 0.035 | 0.162 | 0.922 | 1.237 | 4.411 | 0.249 | 0.824 | 0.011 | 0.029 | 0.098 | 0.329 |
| MS-206 | 0.010 | 0.035 | 0.161 | 1.142 | 0.986 | 3.484 | 0.225 | 0.614 | 0.009 | 0.024 | 0.095 | 0.272 |
| MS-207 | 0.010 | 0.036 | 0.252 | 0.840 | 1.285 | 5.873 | 0.240 | 0.836 | 0.026 | 0.115 | 0.211 | 0.875 |
| MS-208 | 0.007 | 0.036 | 0.699 | 5.174 | 3.569 | 17.98 | 0.206 | 1.018 | 0.016 | 0.047 | 0.231 | 0.816 |
| MS-209 | 0.007 | 0.025 | 0.276 | 1.833 | 1.155 | 5.751 | 0.174 | 0.618 | 0.009 | 0.029 | 0.088 | 0.303 |
| MS-210 | 0.005 | 0.013 | 0.091 | 0.594 | 0.598 | 2.834 | 0.097 | 0.340 | 0.003 | 0.013 | 0.044 | 0.156 |
| MS-211 | 0.008 | 0.028 | 0.237 | 1.514 | 1.151 | 4.240 | 0.205 | 0.744 | 0.010 | 0.025 | 0.092 | 0.309 |
| MS-212 | 0.009 | 0.041 | 0.304 | 1.938 | 1.395 | 6.928 | 0.242 | 1.130 | 0.007 | 0.031 | 0.113 | 0.399 |
| MS-213 | 0.007 | 0.028 | 0.183 | 1.271 | 0.955 | 5.049 | 0.141 | 0.689 | 0.011 | 0.033 | 0.119 | 0.315 |
| MS-214 | 0.005 | 0.018 | 0.221 | 1.553 | 0.987 | 4.567 | 0.185 | 0.630 | 0.009 | 0.024 | 0.090 | 0.238 |
| MS-215 | 0.007 | 0.023 | 0.157 | 1.118 | 1.174 | 5.438 | 0.224 | 0.760 | 0.011 | 0.031 | 0.095 | 0.305 |
| MS-216 | 0.007 | 0.020 | 0.132 | 0.887 | 1.116 | 4.999 | 0.194 | 0.517 | 0.009 | 0.032 | 0.084 | 0.277 |
| MS-217 | 0.010 | 0.037 | 0.260 | 1.678 | 1.159 | 5.397 | 0.216 | 0.743 | 0.010 | 0.038 | 0.075 | 0.275 |
| MS-218 | 0.005 | 0.018 | 0.142 | 1.016 | 0.815 | 4.195 | 0.156 | 0.557 | 0.010 | 0.027 | 0.091 | 0.312 |
| MS-219 | 0.006 | 0.020 | 0.308 | 1.349 | 0.966 | 3.559 | 0.176 | 0.609 | 0.007 | 0.022 | 0.104 | 0.278 |
| MS-220 | 0.009 | 0.027 | 0.215 | 1.023 | 1.242 | 4.415 | 0.193 | 0.528 | 0.009 | 0.027 | 0.099 | 0.335 |
| MS-224 | 0.005 | 0.022 | 0.269 | 1.219 | 1.147 | 4.026 | 0.162 | 0.564 | 0.009 | 0.032 | 0.096 | 0.329 |
| MS-221 | 0.008 | 0.025 | 0.096 | 0.652 | 1.147 | 4.026 | 0.186 | 0.510 | 0.010 | 0.024 | 0.112 | 0.361 |
| MS-222 | 0.007 | 0.022 | 0.089 | 0.622 | 0.767 | 4.198 | 0.104 | 0.801 | 0.007 | 0.023 | 0.077 | 0.257 |
| MS-223 | 0.008 | 0.025 | 0.180 | 0.848 | 1.386 | 4.967 | 0.206 | 0.705 | 0.011 | 0.030 | 0.092 | 0.324 |
| Control | 0.012 | 0.042 | 0.242 | 1.292 | 1.226 | 3.951 | 0.256 | 0.706 | 0.004 | 0.019 | 0.045 | 0.195 |
| MS-257 | 0.026 | 0.082 | 0.469 | 2.447 | 1.832 | 6.498 | 0.455 | 1.516 | 0.012 | 0.050 | 0.123 | 0.561 |
| MS-258 | 0.026 | 0.082 | 0.336 | 1.839 | 2.097 | 7.250 | 0.459 | 1.506 | 0.016 | 0.061 | 0.146 | 0.393 |
| MS-259 | 0.030 | 0.091 | 0.364 | 1.912 | 2.080 | 7.150 | 0.422 | 1.912 | 0.016 | 0.061 | 0.135 | 0.617 |
| MS-260 | 0.025 | 0.077 | 0.459 | 1.520 | 1.834 | 6.213 | 0.443 | 1.185 | 0.009 | 0.038 | 0.081 | 0.369 |

**Supplemental Table 4:** Analytical characterization of round 2 variants.

| **Molecule Set** | **SEC (% monomer)** | **SEC (% dimer)** | **SEC (% Oligomer)** | **DSF T1 °C (Avg. n=2)** | **DSF T1**  **(Std Dev)** | **DSF T2 °C (Avg. n=2)** | **DSF T2**  **(Std Dev)** |
| --- | --- | --- | --- | --- | --- | --- | --- |
| MS-194 | 92.40 | 3.72 | 3.89 | 70.00 | 0.01 |  |  |
| MS-225 | 89.07 | 3.12 | 7.81 | 69.44 | 0.05 |  |  |
| MS-226 | 93.49 | 3.51 | 3.00 | 69.52 | 0.03 |  |  |
| MS-227 | 90.18 | 3.10 | 6.72 | 69.76 | 0.07 |  |  |
| MS-228 | 95.37 | 3.59 | 1.04 | 70.48 | 0.02 | 74.50 | 0.00 |
| MS-229 | 93.39 | 4.03 | 2.58 | 70.04 | 0.05 |  |  |
| MS-230 | 94.00 | 3.42 | 2.58 | 70.25 | 0.05 |  |  |
| MS-231 | 95.35 | 3.87 | 0.79 | 70.41 | 0.02 | 74.50 | 0.03 |
| MS-232 | 94.62 | 3.52 | 1.86 | 70.07 | 0.03 |  |  |
| MS-233 | 95.45 | 3.94 | 0.61 | 70.30 | 0.00 | 76.69 | 0.15 |
| MS-234 | 96.70 | 2.94 | 0.36 | 70.40 | 0.06 | 76.85 | 0.11 |
| MS-235 | 92.77 | 3.46 | 3.77 | 69.74 | 0.05 |  |  |
| MS-236 | 93.06 | 3.78 | 3.16 | 69.92 | 0.10 |  |  |
| MS-237 | 94.61 | 4.03 | 1.36 | 70.47 | 0.02 | 74.50 | 0.00 |
| MS-238 | 91.73 | 3.94 | 4.33 | 69.36 | 0.01 |  |  |
| MS-200 | 94.64 | 4.56 | 0.80 | 70.15 | 0.01 | 74.62 | 0.33 |
| MS-239 | 95.99 | 3.35 | 0.66 | 70.28 | 0.01 | 75.42 | 0.09 |
| MS-240 | 94.87 | 3.61 | 1.52 | 69.99 | 0.01 |  |  |
| MS-241 | 94.36 | 4.93 | 0.71 | 70.11 | 0.02 | 76.41 | 0.04 |
| MS-201 | 94.58 | 4.68 | 0.73 | 70.28 | 0.04 | 76.69 | 0.01 |
| MS-242 | 94.82 | 4.52 | 0.66 | 70.25 | 0.02 | 77.30 | 0.03 |
| MS-243 | 91.81 | 4.51 | 3.67 | 69.57 | 0.06 |  |  |
| MS-244 | 93.97 | 5.04 | 0.99 | 70.17 | 0.02 | 74.70 | 0.18 |
| MS-202 | 93.88 | 5.04 | 1.08 | 70.42 | 0.00 | 75.43 | 0.15 |
| MS-245 | 93.40 | 5.61 | 0.99 | 70.36 | 0.02 | 76.15 | 0.04 |
| MS-246 | 94.88 | 4.59 | 0.53 | 70.19 | 0.05 | 77.13 | 0.09 |
| MS-247 | 94.37 | 4.88 | 0.75 | 69.44 | 0.05 |  |  |

**Supplemental Table 5:** Round 2 biophysical characteristics for the combination variants

| **Molecule Set** | **Shoulder Score (Avg. n=2)** | **(Std Dev)** | **Inflection Pt of Unfolding (Avg n=3)** | **Std Dev** | **pH 3.3 HMW% (Avg n=2)** | **Std Dev** | **PEG solubility (avg. n=2)** | **Std Dev** |
| --- | --- | --- | --- | --- | --- | --- | --- | --- |
| MS-194 | 7.65 | 0.17 | 2.44 | 0.01 | 39.76 | 0.19 | 0.14 | 0.02 |
| MS-225 | 7.22 | 0.97 | 2.37 | 0.03 | 40.43 | 14.01 | 0.18 | 0.02 |
| MS-226 | 7.72 | 0.16 | 2.49 | 0.04 | 14.33 | 6.02 | 0.19 | 0.01 |
| MS-227 | 8.12 | 0.61 | 2.53 | 0.03 | 28.13 | 14.86 | 0.18 | 0.02 |
| MS-228 | 16.25 | 0.55 | 2.64 | 0.02 | 9.10 | 6.29 | 0.17 | 0.02 |
| MS-229 | 9.03 | 0.98 | 2.60 | 0.01 | 9.77 | 2.43 | 0.19 | 0.02 |
| MS-230 | 10.03 | 0.39 | 2.62 | 0.05 | 12.14 | 2.04 | 0.18 | 0.02 |
| MS-231 | 20.80 | 0.22 | 2.63 | 0.02 | 3.35 | 0.16 | 0.14 | 0.02 |
| MS-232 | 9.70 | 0.25 | 2.65 | 0.06 | 8.67 | 1.77 | 0.19 | 0.01 |
| MS-233 | 20.00 | 0.23 | 2.98 | 0.03 | 2.48 | 0.03 | 0.17 | 0.02 |
| MS-234 | 28.36 | 2.18 | 2.97 | 0.19 | 2.61 | 0.51 | 0.15 | 0.02 |
| MS-235 | 7.79 | 0.22 | 2.60 | 0.03 | 13.92 | 1.48 | 0.19 | 0.01 |
| MS-236 | 8.32 | 0.07 | 2.53 | 0.00 | 22.13 | 6.12 | 0.19 | 0.02 |
| MS-237 | 16.01 | 0.05 | 2.65 | 0.05 | 4.58 | 0.49 | 0.15 | 0.03 |
| MS-238 | 8.21 | 0.56 | 2.57 | 0.01 | 31.37 | 25.30 | 0.20 | 0.01 |
| MS-200 | 16.12 | 0.42 | 2.93 | 0.04 | 2.93 | 0.01 | 0.19 | 0.02 |
| MS-239 | 22.28 | 0.72 | 2.98 | 0.04 | 3.19 | 0.49 | 0.18 | 0.02 |
| MS-240 | 9.98 | 0.18 | 2.71 | 0.03 | 9.63 | 4.33 | 0.19 | 0.02 |
| MS-241 | 21.39 | 0.09 | 2.98 | 0.06 | 3.12 | 0.06 | 0.17 | 0.03 |
| MS-201 | 29.39 | 0.45 | 3.15 | 0.03 | 2.21 | 0.06 | 0.17 | 0.02 |
| MS-242 | 23.85 | 0.29 | 2.89 | 0.14 | 2.49 | 0.06 | 0.19 | 0.02 |
| MS-243 | 7.62 | 0.22 | 2.57 | 0.03 | 9.86 | 1.38 | 0.18 | 0.01 |
| MS-244 | 16.12 | 0.51 | 2.90 | 0.08 | 3.25 | 0.16 | 0.18 | 0.02 |
| MS-202 | 22.49 | 1.53 | 3.08 | 0.09 | 3.64 | 0.09 | 0.17 | 0.02 |
| MS-245 | 18.77 | 0.55 | 3.06 | 0.07 | 3.29 | 0.30 | 0.18 | 0.02 |
| MS-246 | 22.39 | 0.97 | 3.29 | 0.03 | 2.32 | 0.23 | 0.17 | 0.03 |
| MS-247 | 7.22 | 0.97 | 2.91 | 0.07 | 3.14 | 0.24 | 0.19 | 0.02 |

**Supplemental Table 6:** Neutralization analysis of selected round 2 variants in TZM.bl cells. Loss of potency are values > 3-fold of control value. Assay Set up: mAbs tested at primary concentration of 25 ug/ml and titrated 5-fold 7x (duplicate wells)

|  | **SC422661.8** | | **WITO4160.33** | | **CAAN5342.A2** | | **DU156.12** | | **DU172.17** | | **CNE17** | |
| --- | --- | --- | --- | --- | --- | --- | --- | --- | --- | --- | --- | --- |
| **Molecule Set** | **IC50** | **IC80** | **IC50** | **IC80** | **IC50** | **IC80** | **IC50** | **IC80** | **IC50** | **IC80** | **IC50** | **IC80** |
| Control | 0.045 | 0.157 | 0.205 | 1.439 | 0.005 | 0.019 | 0.008 | 0.034 | 0.055 | 0.156 | 1.34 | 4.574 |
| MS-228 | 0.03 | 0.114 | 0.097 | 0.71 | 0.002 | 0.01 | 0.003 | 0.017 | 0.036 | 0.134 | 0.928 | 3.21 |
| MS-231 | 0.032 | 0.092 | 0.092 | 0.646 | 0.001 | 0.012 | 0.003 | 0.017 | 0.034 | 0.125 | 0.796 | 2.719 |
| MS-233 | 0.037 | 0.128 | 0.172 | 0.789 | 0.005 | 0.015 | 0.004 | 0.02 | 0.033 | 0.124 | 0.625 | 3.085 |
| MS-234 | 0.04 | 0.136 | 0.22 | 0.932 | 0.003 | 0.011 | 0.004 | 0.017 | 0.058 | 0.232 | 0.734 | 2.646 |
| MS-237 | 0.028 | 0.126 | 0.113 | 0.744 | 0.004 | 0.016 | 0.004 | 0.019 | 0.037 | 0.117 | 0.792 | 3.673 |
| MS-200 | 0.036 | 0.156 | 0.13 | 0.794 | 0.005 | 0.02 | 0.007 | 0.028 | 0.037 | 0.152 | 0.922 | 3.21 |
| MS-239 | 0.033 | 0.114 | 0.199 | 0.951 | 0.003 | 0.012 | 0.005 | 0.017 | 0.03 | 0.125 | 0.63 | 2.887 |
| MS-241 | 0.034 | 0.106 | 0.229 | 0.999 | 0.004 | 0.014 | 0.008 | 0.02 | 0.038 | 0.137 | 0.81 | 2.939 |
| MS-201 | 0.027 | 0.104 | 0.177 | 1.153 | 0.002 | 0.011 | 0.005 | 0.016 | 0.028 | 0.102 | 0.782 | 2.62 |
| MS-242 | 0.04 | 0.116 | 0.145 | 0.889 | 0.003 | 0.012 | 0.006 | 0.021 | 0.034 | 0.161 | 0.762 | 3.331 |
| MS-244 | 0.041 | 0.123 | 0.172 | 1.169 | 0.005 | 0.017 | 0.003 | 0.017 | 0.045 | 0.167 | 0.694 | 3.637 |
| MS-202 | 0.028 | 0.135 | 0.185 | 0.708 | 0.003 | 0.011 | 0.004 | 0.021 | 0.028 | 0.104 | 0.825 | 2.903 |
| MS-245 | 0.029 | 0.102 | 0.132 | 0.774 | 0.002 | 0.012 | 0.006 | 0.022 | 0.038 | 0.139 | 0.991 | 4.443 |
| MS-246 | 0.037 | 0.128 | 0.145 | 0.82 | 0.004 | 0.017 | 0.006 | 0.025 | 0.039 | 0.146 | 0.907 | 3.175 |
| MS-247 | 0.034 | 0.151 | 0.107 | 0.611 | 0.003 | 0.014 | 0.009 | 0.032 | 0.03 | 0.149 | 0.642 | 3.141 |

|  | **CNE30** | | **CNE53** | | **235-47** | | **X1193_c1** | | **X1254_c3** | | **3301.v1.c24** | |
| --- | --- | --- | --- | --- | --- | --- | --- | --- | --- | --- | --- | --- |
| **Molecule Set** | **IC50** | **IC80** | **IC50** | **IC80** | **IC50** | **IC80** | **IC50** | **IC80** | **IC50** | **IC80** | **IC50** | **IC80** |
| Control | 0.258 | 0.709 | 0.017 | 0.049 | 0.029 | 0.116 | 0.04 | 0.139 | 0.055 | 0.154 | 0.008 | 0.021 |
| MS-228 | 0.215 | 0.595 | 0.007 | 0.028 | 0.019 | 0.086 | 0.03 | 0.13 | 0.039 | 0.117 | 0.003 | 0.013 |
| MS-231 | 0.184 | 0.517 | 0.007 | 0.025 | 0.016 | 0.078 | 0.028 | 0.12 | 0.035 | 0.116 | 0.002 | 0.012 |
| MS-233 | 0.186 | 0.649 | 0.006 | 0.023 | 0.03 | 0.113 | 0.03 | 0.133 | 0.041 | 0.129 | 0.003 | 0.016 |
| MS-234 | 0.184 | 0.5 | 0.008 | 0.037 | 0.019 | 0.077 | 0.031 | 0.142 | 0.03 | 0.122 | 0.003 | 0.014 |
| MS-237 | 0.17 | 0.46 | 0.005 | 0.027 | 0.022 | 0.086 | 0.042 | 0.146 | 0.035 | 0.101 | 0.002 | 0.014 |
| MS-200 | 0.17 | 0.583 | 0.007 | 0.027 | 0.022 | 0.085 | 0.059 | 0.177 | 0.047 | 0.133 | 0.005 | 0.019 |
| MS-239 | 0.175 | 0.588 | 0.004 | 0.02 | 0.023 | 0.088 | 0.03 | 0.182 | 0.036 | 0.102 | 0.002 | 0.011 |
| MS-241 | 0.181 | 0.498 | 0.006 | 0.023 | 0.028 | 0.08 | 0.042 | 0.155 | 0.034 | 0.119 | 0.003 | 0.01 |
| MS-201 | 0.173 | 0.471 | 0.003 | 0.025 | 0.011 | 0.067 | 0.033 | 0.154 | 0.037 | 0.1 | 0.003 | 0.008 |
| MS-242 | 0.197 | 0.535 | 0.004 | 0.023 | 0.028 | 0.092 | 0.042 | 0.185 | 0.031 | 0.107 | 0.003 | 0.009 |
| MS-244 | 0.18 | 0.639 | 0.001 | 0.014 | 0.032 | 0.113 | 0.041 | 0.184 | 0.027 | 0.108 | 0.001 | 0.005 |
| MS-202 | 0.16 | 0.568 | 0.005 | 0.021 | 0.022 | 0.084 | 0.037 | 0.139 | 0.028 | 0.117 | 0.005 | 0.018 |
| MS-245 | 0.156 | 0.553 | 0.004 | 0.019 | 0.028 | 0.1 | 0.049 | 0.172 | 0.04 | 0.137 | 0.006 | 0.019 |
| MS-246 | 0.204 | 0.557 | 0.002 | 0.021 | 0.037 | 0.128 | 0.046 | 0.199 | 0.047 | 0.164 | 0.007 | 0.021 |
| MS-247 | 0.197 | 0.554 | 0.005 | 0.027 | 0.036 | 0.126 | 0.043 | 0.203 | 0.029 | 0.113 | 0.008 | 0.023 |

**Supplemental Table 7:** Individual-level PK parameters derived from the estimated two-compartment population models for each 10-1074 variant. The model for the JB-1 variant excluded data for animal ID 9 after day 10 due to a potential ADA response.

| 10-1074 variant | Animal ID | Vc | CL | Q | Vp |
| --- | --- | --- | --- | --- | --- |
| Parental | 1 | 6.144 | 5.27 | 1.109 | 3.591 |
|  | 2 | 6.144 | 5.896 | 1.109 | 2.655 |
|  | 3 | 6.144 | 5.83 | 1.109 | 3.12 |
|  | 4 | 6.144 | 5.578 | 1.109 | 3.661 |
|  | Median (range) | 6.14 (6.14 - 6.14) | 5.70 (5.27 - 5.9) | 1.11 (1.11 - 1.11) | 3.36 (2.66 - 3.66) |
| MS-194 | 5 | 3.305 | 0.631 | 3.364 | 12.332 |
|  | 6 | 3.305 | 0.596 | 3.364 | 11.575 |
|  | 7 | 3.305 | 0.591 | 3.364 | 9.691 |
|  | 8 | 3.305 | 0.696 | 3.364 | 4.986 |
|  | Median (range) | 3.31 (3.31 - 3.31) | 0.61 (0.59 - 0.7) | 3.36 (3.36 - 3.36) | 10.63 (4.99 - 12.33) |
| MS-200 | 9 | 4.791 | 1.258 | 4.307 | 7.308 |
|  | 10 | 4.791 | 1.272 | 4.307 | 6.313 |
|  | 11 | 4.791 | 1.228 | 4.307 | 10.234 |
|  | 12 | 4.791 | 1.24 | 4.307 | 7.296 |
|  | Median (range) | 4.79 (4.79 - 4.79) | 1.25 (1.23 - 1.27) | 4.31 (4.31 - 4.31) | 7.30 (6.31 - 10.23) |
| MS-201 | 13 | 5.801 | 1.079 | 0.704 | 6.102 |
|  | 14 | 5.801 | 0.993 | 1.612 | 6.102 |
|  | 15 | 5.801 | 1.306 | 3.342 | 6.101 |
|  | 16 | 5.801 | 1.476 | 4.894 | 6.101 |
|  | Median (range) | 5.80 (5.8 - 5.8) | 1.19 (0.99 - 1.48) | 2.48 (0.7 - 4.89) | 6.10 (6.1 - 6.1) |
| MS-202 | 17 | 8.977 | 0.963 | 1.59 | 19.386 |
|  | 18 | 8.1 | 0.964 | 1.59 | 19.387 |
|  | 19 | 8.113 | 0.964 | 1.59 | 19.387 |
|  | 20 | 9.152 | 0.963 | 1.59 | 19.386 |
|  | Median (range) | 8.55 (8.1 - 9.15) | 0.96 (0.96 - 0.96) | 1.59 (1.59 - 1.59) | 19.39 (19.39 - 19.39) |

**Supplemental Table 8:** Estimated concentration curves by animal using two-compartment population PK models for each antibody group excluding all values after the first concentration below the LLoQ. Points denote observed data used in the model and the lines denote concentration curves estimated using individual-level parameter estimates from the population models. The model for the JB-1 variant excluded data for animal ID 9 after day 10 due to a potential anti-drug antibody response.


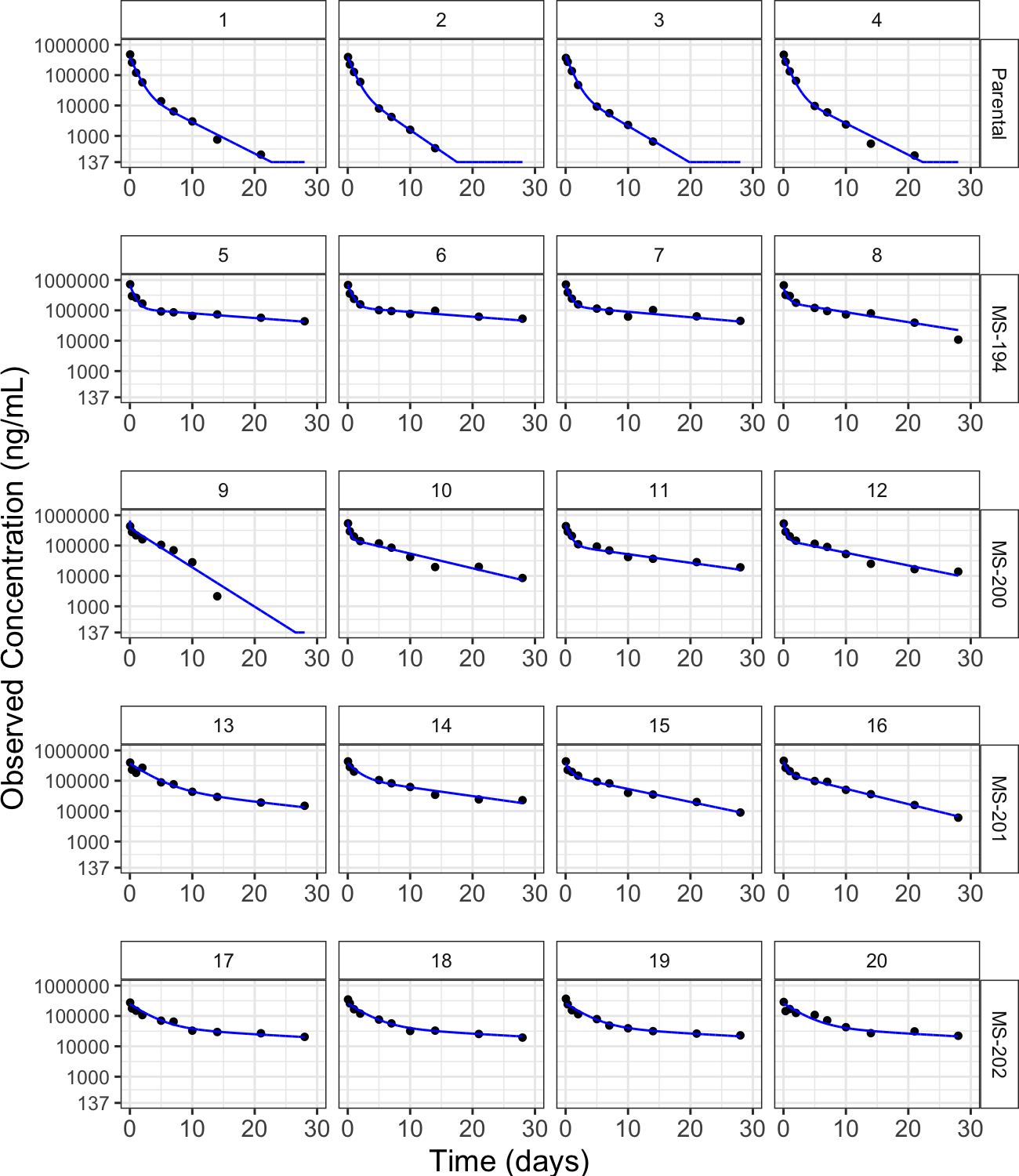


**Supplemental Table 9:** Two-sided group comparisons of half-life estimates using the Mann-Whitney U Test.

| Comparison | Median (Range) | p-value |
| --- | --- | --- |
| Parental (-LS) vs. MS-194 | 2.69 (2.13 - 2.91) vs. 17.99 (8.9 - 19.23) | 0.0105 |
| Parental (-LS) vs. MS-200 | 2.69 (2.13 - 2.91) vs. 7.47 (6.67 - 9.67) | 0.0105 |
| Parental (-LS) vs. MS-201 | 2.69 (2.13 - 2.91) vs. 8.44 (6.06 - 11.75) | 0.0105 |
| Parental (-LS) vs. MS-202 | 2.69 (2.13 - 2.91) vs. 26.59 (26.36 - 26.92) | 0.0105 |
| MS-194 vs. MS-200 | 17.99 (8.9 - 19.23) vs. 7.47 (6.67 - 9.67) | 0.0433 |
| MS-194 vs. MS-201 | 17.99 (8.9 - 19.23) vs. 8.44 (6.06 - 11.75) | 0.0833 |
| MS-194 vs. MS-202 | 17.99 (8.9 - 19.23) vs. 26.59 (26.36 - 26.92) | 0.0209 |
| MS-200 vs. MS-201 | 7.47 (6.67 - 9.67) vs. 8.44 (6.06 - 11.75) | 0.7728 |
| MS-200 vs. MS-202 | 7.47 (6.67 - 9.67) vs. 26.59 (26.36 - 26.92) | 0.0209 |
| MS-201 vs. MS-202 | 8.44 (6.06 - 11.75) vs. 26.59 (26.36 - 26.92) | 0.0209 |
